# The rice *CYP78A* gene *BSR2* confers resistance to *Rhizoctonia solani* and affects seed size and growth in *Arabidopsis* and rice

**DOI:** 10.1038/s41598-018-37365-1

**Published:** 2019-01-24

**Authors:** Satoru Maeda, Joseph G. Dubouzet, Youichi Kondou, Yusuke Jikumaru, Shigemi Seo, Kenji Oda, Minami Matsui, Hirohiko Hirochika, Masaki Mori

**Affiliations:** 1 Institute of Agrobiological Sciences, NARO (NIAS), Tsukuba, Japan; 2 RIKEN Yokohama, Tsurumi, Yokohama Japan; 3 Research Institute for Biological Sciences, Okayama Prefectural Technology Center for Agriculture, Forestry and Fisheries, Okayama, Japan

## Abstract

The fungal pathogen *Rhizoctonia solani* causes devastating diseases in hundreds of plant species. Among these, *R. solani* causes sheath blight, one of the three major diseases in rice. To date, few genes have been reported that confer resistance to *R. solani*. Here, rice-FOX *Arabidopsis* lines identified as having resistance to a bacterial pathogen, *Pseudomonas syringae* pv*. tomato* DC3000, and a fungal pathogen, *Colletotrichum higginsianum* were screened for disease resistance to *R. solani*. *BROAD-SPECTRUM RESISTANCE2* (*BSR2*), a gene encoding an uncharacterized cytochrome P450 protein belonging to the CYP78A family, conferred resistance to *R. solani* in *Arabidopsis*. When overexpressed in rice, *BSR2* also conferred resistance to two *R. solani* anastomosis groups. Both *Arabidopsis* and rice plants overexpressing *BSR2* had slower growth and produced longer seeds than wild-type control plants. In contrast, *BSR2*-knockdown rice plants were more susceptible to *R. solani* and displayed faster growth and shorter seeds in comparison with the control. These results indicate that *BSR2* is associated with disease resistance, growth rate and seed size in rice and suggest that its function is evolutionarily conserved in both monocot rice and dicot *Arabidopsis*.

## Introduction

*Rhizoctonia solani*, belonging to the sub-division Basidiomycota, is a globally distributed necrotrophic soil-borne fungus. The host-range of *R. solani* spans numerous plant families including, but not limited to, wheat, rice, barley, canola, soybean, corn, potato and sugar beet^1^. *R. solani* colonizes different plant organs depending on the plant species; for example, leaves are infected in rice, whereas roots are infected in plants such as soybean, sugar beet and tomato. Because *R. solani* produces basidiospores under certain conditions, the fungus can be subdivided into anastomosis groups (AGs) based on hyphal anastomosis and physiological-biochemical characteristics^2^. To date, isolates of *R. solani* from plant species have been assigned to 13 AGs (AG1 to AG13), some of which include several subgroups^3,4^. Rice sheath blight, one of the major three diseases of rice, is caused by *R. solani* AG-1 IA and rice brown sheath blight is caused by *R. solani* AG-2-2 IIIB.

Despite the severe damage caused by *R. solani*, conventional breeding has had limited efficacy in introducing genetic resistance to *R. solani* in any crop species^5^. There is no rice cultivar that is fully resistant to *R. solani*; therefore, it is difficult to breed rice resistance to sheath blight using conventional breeding and selection methods. Genetic overexpression of several rice genes have been shown to confer resistance to *R. solani*, including *OsACS2*^6^, *Os2H16*^7^, *OsWRKYs*^8–10^ and *OsASR2*^11^. The transcription factors OsWRKY80 and OsASR2 positively regulate OsWRKY4 and Os2H16, respectively^8,11^.

To date, several loss-of-function and gain-of-function mutant lines have been developed to isolate and investigate the functions of genes in rice^12,13^. Because approximately 30% of rice genes are predicted to be members of clustered and redundant gene families^14^, it can be difficult, if not impossible, to determine their functions using loss-of-function (silencing) approaches. In contrast, gain-of-function approaches enable direct screening of phenotypes of interest such as disease resistance.

Ichikawa *et al*.^15^ developed the Full-length cDNA OvereXpressing (FOX)-hunting system as an alternative to activation-tagging in *Arabidopsis*. In this system, transcriptomes of full-length cDNAs from another plant species are ectopically expressed in *Arabidopsis*. The short lifespan and small stature of *Arabidopsis* allow rapid screening for desirable transgenic phenotypes using this approach. A rice-FOX *Arabidopsis* population of 23,000 lines was previously generated by introducing 13,000 full-length rice cDNAs under the control of the CaMV 35S promoter into *Arabidopsis* ecotype Columbia^16^. Several screenings have been performed on these lines to date, and genes related to heat stress tolerance (*OsHsfA2e*^17^), salt tolerance (*OsSMCP1*^18^; *OsNAC063*^19^; *OsCEST*^20^ and *JAmyb*^21^), secondary metabolism (*OsLBD37*^22^), disease resistance (*BSR1*^23^) and photosynthesis (*FNR1/FNR2*^24^) have been identified.

We previously screened 20,000 rice-FOX *Arabidopsis* lines for resistance to a bacterial pathogen, *Pseudomonas syringae* pv*. tomato* DC3000 (*Pst* DC3000), and then a fungal pathogen, *Colletotrichum higginsianum*, to find rice genes conferring broad-spectrum disease resistance^23^. We identified *BROAD-SPECTRUM RESISTANCE1* (*BSR1*), a gene encoding a receptor-like cytoplasmic kinase. *BSR1* conferred resistance to *Pst* DC3000 and *C. higginsianum* when overexpressed in *Arabidopsis*, and to *Xanthomonas oryzae, Pyricularia oryzae* (syn. *Magnaporthe oryzae*), *Burkholderia glumae*, and *Cochliobolus miyabeanus* when overexpressed in rice^23,25^. However, neither *BSR1-*overexpression (*BSR1-*OX) *Arabidopsis* nor rice displayed resistance to *R. solani*. To identify genes conferring resistance to *R. solani* from rice, we hypothesized that some of the other FOX lines showing resistance to both *Pst* DC3000 and fungal *C. higginsianum* might also display resistance to the fungus *R. solani*, even though *Colletotrichum* ascomycetes and the basidiomycete *R. solani* are not closely related according to their taxonomic classification. We decided to screen FOX lines showing resistance to both *Pst* DC3000 and *C. higginsianum* against *R. solani*.

In this study, before starting *R. solani* screening, we conducted *Pst* DC3000 screening using an additional 1,000 Rice-FOX *Arabidopsis* lines to increase the number of candidate resistant lines. By rescreening the FOX lines showing resistance to both *Pst* DC3000 and *C. higginsianum*, we succeeded in identifying a novel P450 gene, named *BROAD-SPECTRUM RESISTANCE2* (*BSR2*). *BSR2* conferred resistance to *R. solani* in both *Arabidopsis* and rice. *BSR2* was ultimately found from the additional 1,000 lines that were screened. Surprisingly, both *BSR2*-OX *Arabidopsis* and rice displayed slower growth and longer seeds in comparison with the wild-type (WT). Our findings suggest that *BSR2* is involved in pleiotropic functions in the monocot rice and strongly suggest that the molecular mechanism underlying these fundamental functions is conserved in the dicot *Arabidopsis* as well.

## Results

### Screening of additional FOX lines for resistance to *Pst* DC3000

**Figure 1:**
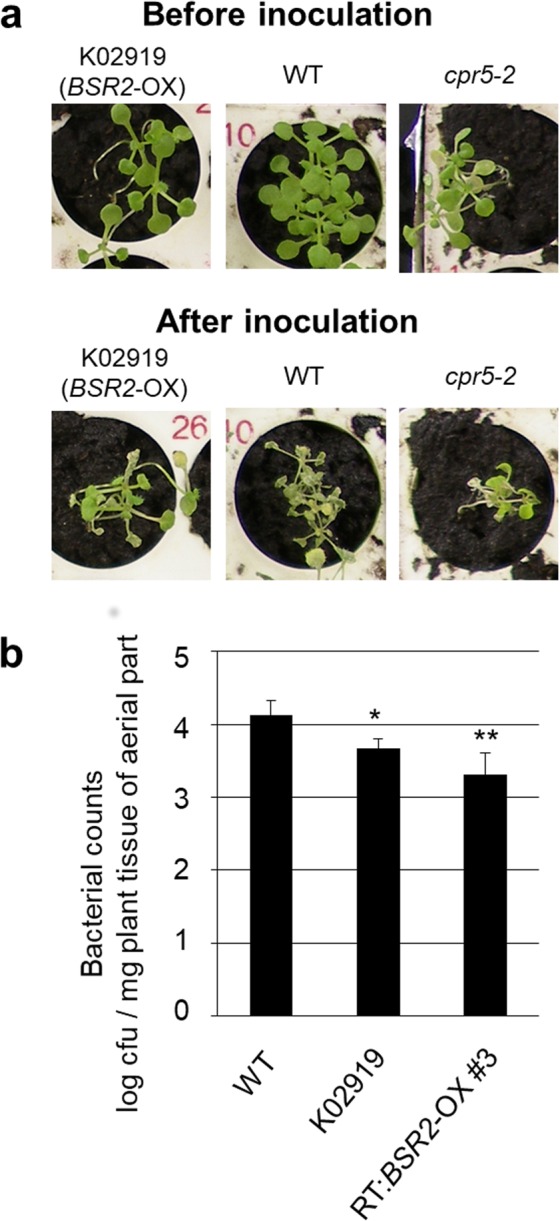
Resistance to *Pst* DC3000 in *BSR2*-OX *Arabidopsis* lines. (**a**) Phenotypic responses to *Pst* DC3000 inoculation. Three-week-old plants were inoculated with 2 × 10^8^ cfu/mL of *Pst* DC3000. The wild-type (WT; Col-0) plants died, but the K02919 (a rice-FOX *Arabidopsis* T2 line; *BSR2*-OX) and *cpr5-2* (control mutant resistant to *Pst* DC3000) plants remained green. (**b**) Growth of *Pst* DC3000 bacteria in plants. *Arabidopsis* plants at about the 20-leaf stage were inoculated with *Pst* DC3000 (10^6^ cfu/mL) by dipping, and the numbers of bacteria in the aerial part of the plants were counted after 4 d. The bacteria in 8 replicates were counted and the differences between WT and two independent *BSR2*-OX lines (K02919 and RT:*BSR2*-OX#3) were significant according to a *t*-test (*P < 0.05; **P < 0.01). Error bars represent the standard deviation.

We previously screened 20,000 rice-FOX *Arabidopsis* lines and obtained 72 lines that were resistant to infection by the *Pst* DC3000 bacterium^23^. More than 10 of these lines also showed resistance to infection by the fungus *C. higginsianum*. Here, we screened an additional 1,000 rice-FOX *Arabidopsis* lines and obtained one line, K02919, which showed clear resistance against *Pst*DC3000. All the WT (Col-0) plants were susceptible to *Pst*DC3000 and died, but the K02919 and resistant *cpr5-2* control plants partly survived after inoculation (Fig. 1a). The K02919 line overexpressed a full-length rice cDNA AK072163 (Os08g0547300) that we termed *BSR2*. We generated six independent retransformed *Arabidopsis* lines (RT:*BSR2-*OX#1–#6) that overexpressed AK072163 (Supplementary Fig. S1). The K17903 line, another original FOX line overexpressing AK072163 cDNA, was also used (Supplementary Fig. S1). Because of the low fertility associated with *BSR2* overexpression and the consequent shortage of transgenic seeds, different transgenic lines overexpressing AK072163 cDNA were used in later experiments.

To quantify the bacterial resistance, the bacterial numbers in the inoculated plants were counted. The bacterial counts in the K02919 and RT:*BSR2-*OX#3 plants were significantly lower than those in the WT plants (Fig. 1b) and the *BSR2* transcript levels in RT:*BSR2-*OX#3 plants were higher than those in the K02919 line plants (Supplementary Fig. S1). Overall, the plant bacterial resistance levels correlated well with the *BSR2* transcript levels (Fig. 1b).

### Resistance to *C. higginsianum*

**Figure 2:**
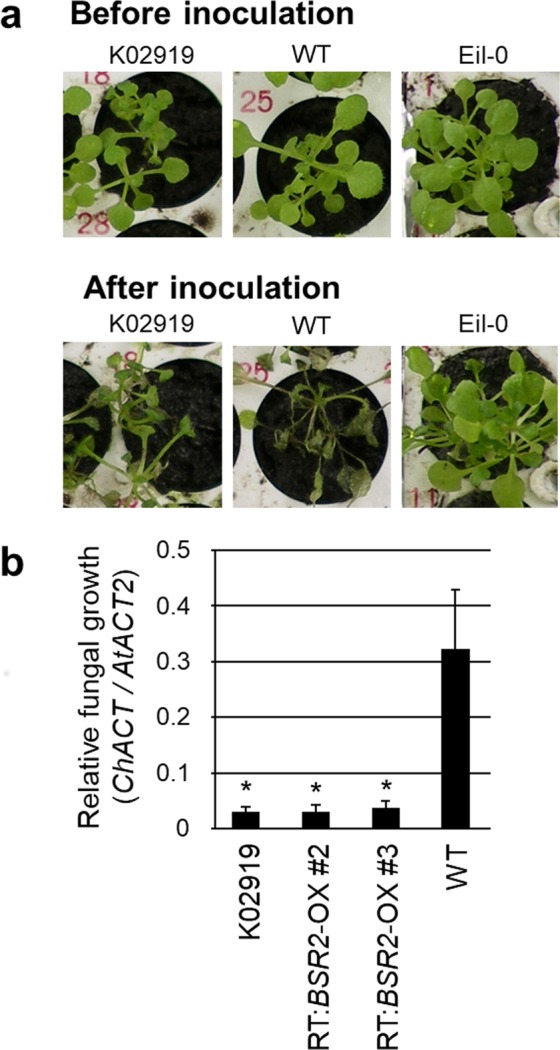
Resistance to *C. higginsianum* in *BSR2*-OX *Arabidopsis* lines. (**a**) Phenotypic responses to *C. higginsianum* inoculation. Three-week-old plants were inoculated with 10^5^ conidia/mL of *C. higginsianum*. Unlike wild-type (WT; Col-0) plants, K02919 (a rice-FOX *Arabidopsis* T2 line; *BSR2*-OX) and Eil-0 (control ecotype resistant to *C. higginsianum*) plants were still green 13 d after inoculation. (**b**) Quantification of the relative fungal growth across plant lines. *C. higginsianum Actin (ACT)* DNA in the *Arabidopsis* plants were measured using quantitative real-time PCR 3 d after inoculation. The amounts of *C. higginsianum* genomic *ACT* DNA relative to *Arabidopsis* genomic *ACT2* DNA in three independent *BSR2*-OX lines (K02919, RT:*BSR2*-OX#2 and RT:*BSR2*-OX#3) were significantly lower than those of WT plants (*P < 0.05 according to a *t*-test; n = 3–5; error bars represent the standard deviation).

To determine whether *BSR2* overexpression is also effective against fungal pathogens, we tested the *BSR2*-OX lines for resistance against the ascomycete *C. higginsianum*. The WT plants completely died after inoculation, but the K02919 and the resistant ecotype (Eil-0) control plants survived (Fig. 2a). For quantitative evaluation, relative fungal growth was compared between the plant lines using qRT-PCR. The relative fungal growths in K02919 and the two retransformed *BSR2*-OX lines (RT:*BSR2*-OX#2 and #3) were significantly lower than those in the WT plants (Fig. 2b).

### Resistance to *R. solani*

*R. solani*, a fungal pathogen classified in the Basidiomycota, causes rice sheath blight, which is one of the three main diseases that devastate rice. We hypothesized that some FOX lines that show resistance to both bacterial *Pst* DC3000 and fungal *C. higginsianum* may also display resistance to the fungal pathogen *R. solani*. In addition to K02919, we previously identified 11 single-cDNA insert rice-FOX *Arabidopsis* lines that had resistance to both *Pst* DC3000 and *C. higginsianum*^23^ as shown in Supplementary Table S1. We further screened these FOX lines to identify the gene that conferred resistance to *R. solani*. We used the *cpr5-2* mutant line with resistance to *Pst* DC3000 and the Eil-0 ecotype line with resistance to *C. higginsianum* as controls.

**Figure 3:**
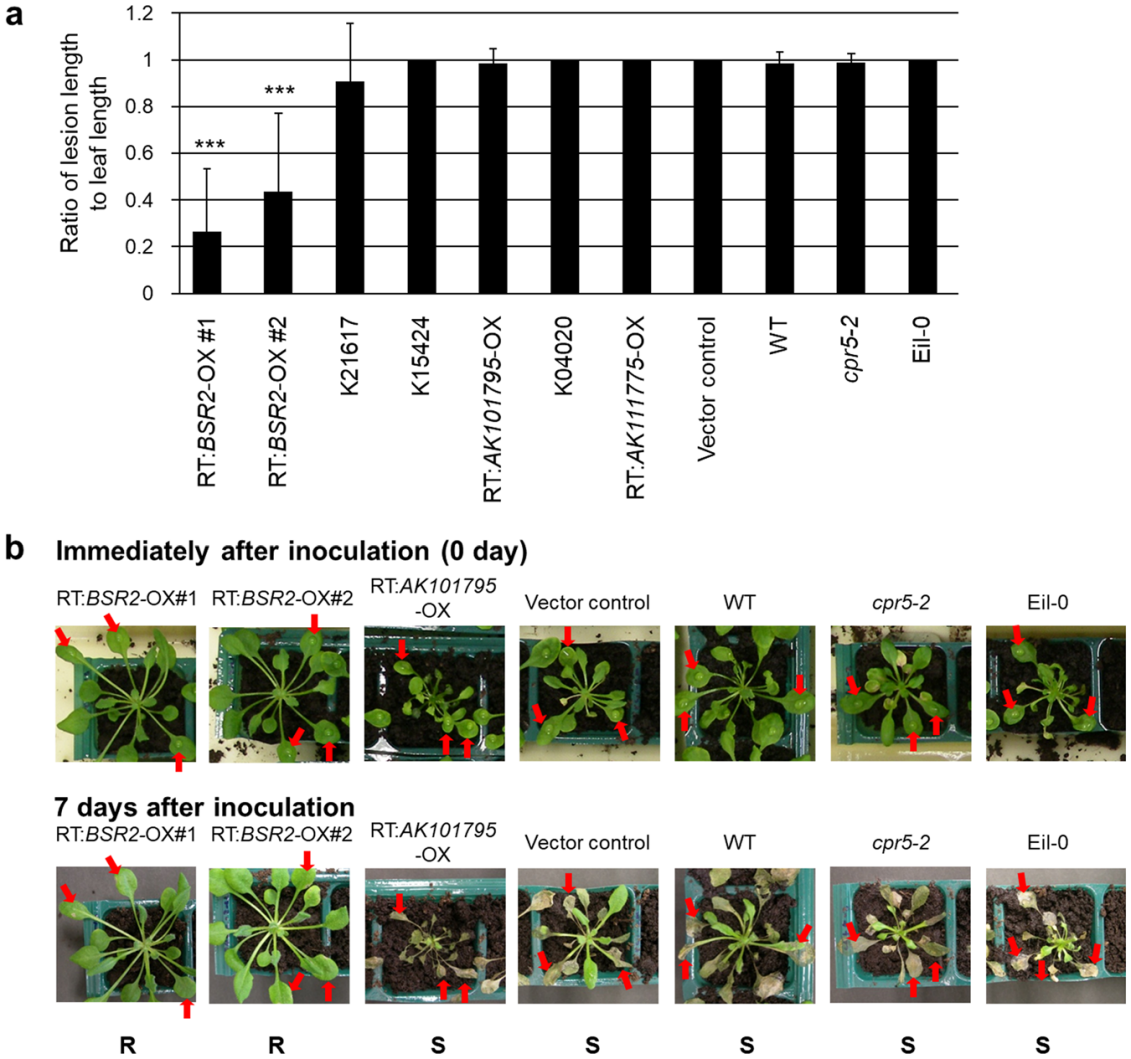
Screening rice-FOX *Arabidopsis* lines for resistance to *R. solani*. (**a**) Evaluation of *R. solani* resistance using the ratio of lesion length to leaf length. Six-week-old plants were used for drop inoculation with *R. solani* (MAFF243956; AG1-1A). Seven rice-FOX *Arabidopsis* lines showing resistance to both *Pst* DC3000 and *C. higginsianum* were used: RT:*BSR2*-OX#1 and #2, RT:AK101795-OX and RT:AK111775-OX are independent retransformed lines for AK072163, AK101795 and AK111775, respectively. K21617, K15424 and K04020 are independent transgenic rice lines with one rice cDNA insert for AK103699, *BSR1* (AK070024) and AK066139, respectively. The lesion length and leaf length were measured 7 d after inoculation. Asterisks indicate that values are significantly different from the WT and vector control plants (***P < 0.001, according to a *t*-test; error bars represent the standard deviation; n = 3). (**b**) Representative *Arabidopsis* plants described in (**a**) immediately after inoculation and 7 d after inoculation. In comparison with the vector control and WT plants, the spread of disease symptoms stopped 7 d after inoculation in the *BSR2*-OX plants (RT:*BSR2*-OX#1 and #2). The arrows indicate the inoculation points. R, resistant; S, susceptible.

Because our main purpose was to identify rice cDNA that are effective against the *R. solani* isolate (MAFF243956; AG-1 IA) that causes rice sheath blight, we used this isolate to screen the FOX lines. This isolate can also infect *Arabidopsis*. Resistance to *R. solani* was evaluated by the ratio of disease lesion length to the drop-inoculated leaf length of six-week-old *Arabidopsis* plants. Most of the *Arabidopsis* lines tested — K21617 (*AK103699*-OX), K15424 (*BSR1*-OX), RT:*AK101795*-OX, K04020 (*AK066139*-OX) and RT:*AK11177*5-OX — were susceptible to *R. solani*. Only two *BSR2*-OX lines (RT:*BSR2*-OX#1 and #2) displayed significant resistance to *R. solani* in comparison with WT, the vector control and other lines (Fig. 3). Therefore, overexpression of *BSR2* conferred resistance to bacterial *Pst* DC3000, fungal *C. higginsianum* and *R. solani* in *Arabidopsis*.

According to the Rice Annotation Project (RAP) database, *BSR2* encodes a cytochrome P450 protein (Supplementary Table S1). The BSR2 protein is classified as a CYP78A15 (CY78C5)-type cytochrome P450^26–28^, although its CYP78A15 sequence has an insertion of three additional amino acids. The sequence alignments and phylogenetic tree for BSR2 and its related P450 proteins are shown in Supplementary Fig. S2.

### Rice resistance to *R. solani* (AG-1 IA) sheath blight

We examined whether overexpression of *BSR2* also confers resistance to sheath blight in rice. The *BSR2* cDNA, AK072163, was inserted downstream of the constitutive maize *ubiquitin* promoter, and this construct was used to generate transgenic rice lines overexpressing *BSR2*. At the organ regeneration step of tissue culture, shoots were produced from *BSR2*-OX calli but roots failed to form on both regeneration and hormone-free media. However, when the rootless regenerated shoots were transferred to soil and watered, roots formed and we were able to use the regenerated plants for subsequent analyses. Overexpression of the *BSR2* cDNA was confirmed by quantitative real-time RT-PCR (qRT-PCR) analysis of the T0 plants (plants regenerated from transgenic calli), and their T1, T2 and T3 seeds were used for the experiments. The *BSR2* expression levels in the *BSR2*-OX transgenic plants are presented in Supplementary Fig. S3. Because of the low fertility associated with *BSR2* overexpression and the consequent shortage of transgenic seeds in rice as well as in *Arabidopsis*, different overexpressing lines were used in later experiments.

**Figure 4:**
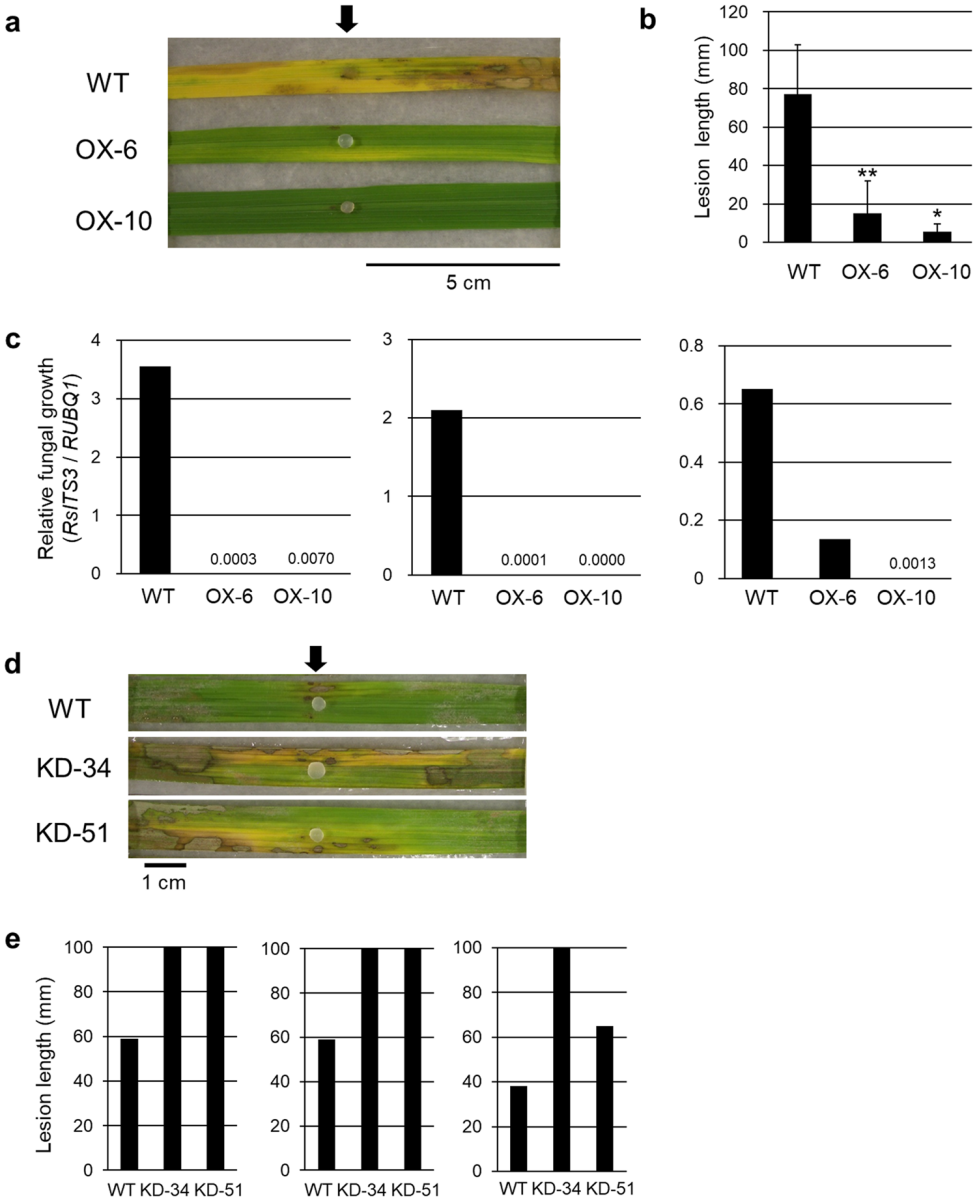
Resistance to *R. solani* (AG-1 IA) sheath blight in *BSR2*-OX and -KD rice lines. Comparisons of (**a**) inoculated leaves, (**b**) lesion lengths and (**c**) relative fungal growth of detached leaf blades from *BSR2*-OX lines 13 d after drop inoculation with *R. solani* (MAFF243956; AG1-1A). The second leaf blades from the top leaf at leaf stages 10–13 were used for inoculation. Asterisks indicate that values are significantly different from the WT (*P < 0.05; **P < 0.01, according to a *t*-test; error bars represent the standard deviation; n = 4). Measurements of relative fungal growth were performed three times with similar results. Comparison of (**d**) inoculated leaves and (**e**) lesion lengths of detached leaf blades of *BSR2*-KD lines 7 d after drop inoculation. The second leaf blades from the top leaf at leaf stages 10–13 were used for inoculation. The lesion lengths in the *BSR2*-KD plants were longer than those in the WT plants. Tests were performed three times with similar results. Arrows indicate the inoculation points.

Leaves cut from the transgenic rice plants were used to evaluate their resistance to *R. solani* (MAFF243956; AG-1 IA). Whereas WT plants developed lesions that extended from the inoculation point, *BSR2*-OX lines showed only restricted lesion formation (Fig. 4a and Supplementary Fig. S4). The lesion lengths in the inoculated *BSR2*-OX leaves were significantly shorter than those in the WT leaves (Figs 4b and S4b). The relative fungal growths were compared between the plant lines using qRT-PCR. The relative fungal growths in two *BSR2*-OX lines were much lower than those in the WT plants in three independent experiments (Fig. 4c). A general sheath inoculation assay was also carried out to evaluate resistance to *R. solani* in whole plants. The lesion lengths of the two *BSR2*-OX lines were significantly shorter than those of the WT plants (Supplementary Fig. S4c). Together, these data confirm that the *BSR2*-OX rice lines have resistance to *R. solani*. Because the results obtained using the detached leaf inoculation assay and the sheath inoculation assay were similar, we used the only detached leaf inoculation assay for subsequent comparisons.

Overexpression of genes sometimes induces artificial effects; therefore, loss-of-function experiments are generally more reliable for determining innate gene functions. To examine whether *BSR2* is innately involved in rice defence against sheath blight, we generated *BSR2*-knockdown (KD) transgenic rice plants that suppress *BSR2* using its 3′-untranslated region (~300 bp) as a trigger. *BSR2* transcript levels in the generated *BSR2*-KD-34, -45 and -51 lines were successfully reduced (Supplementary Fig. S3). The lesion lengths in inoculated *BSR2*-KD rice leaf blades were longer than those in WT rice (Fig. 4d,e and Supplementary Fig. S5), indicating that *BSR2* is innately involved in rice defence against sheath blight caused by *R. solani*.

### Rice resistance to *R. solani* (AG-2-2 IIIB) brown sheath blight

**Figure 5:**
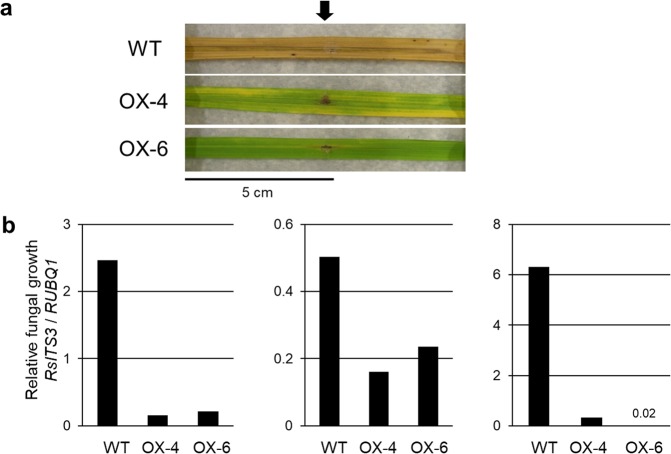
Resistance to *R. solani* (AG-2-2 IIIB) brown sheath blight in *BSR2*-OX rice lines. Comparison of (**a**) inoculated leaves and (**b**) relative fungal growths 11 d after drop inoculation with *R. solani* (Shimomura 9 isolate; AG-2-2 IIIB). The second leaf blades from the flag leaf at the heading stage were used for inoculation. The relative fungal growth levels in the *BSR2*-OX plants were lower than those in the WT plants. Tests were performed three times with similar results. The inoculation points are indicated by an arrow.

We investigated whether overexpression of *BSR2* also conferred resistance to brown sheath blight, another disease that is caused by *R. solani* in rice. For this purpose, we used the *R. solani* Shimomura 9 isolate (AG-2-2 IIIB) instead of *R. solani* (MAFF243956; AG-1 IA). A detached leaf inoculation assay was performed to evaluate the rice resistance to the *R. solani* Shimomura 9 isolate. Whereas WT plants developed lesions that extended from the inoculation point, *BSR2*-OX lines had restricted lesion development (Fig. 5a). For quantitative evaluation, the relative fungal growths were compared using qRT-PCR. The relative fungal growths in the *BSR2*-OX lines were remarkably lower than those in the WT plants in three independent experiments (Fig. 5b). These results indicate that the *BSR2-*OX lines were resistant to brown sheath blight caused by *R. solani* (AG-2-2 IIIB). Therefore, in rice, *BSR2* confers resistance to *R. solani* isolates belonging to two different AGs. To our knowledge, no other gene has been reported to confer resistance to *R. solani* isolates belonging to more than one AG.

### Changes in the growth rate, plant height and reproductive organ size caused by overexpression of *BSR2* in *Arabidopsis*

**Figure 6:**
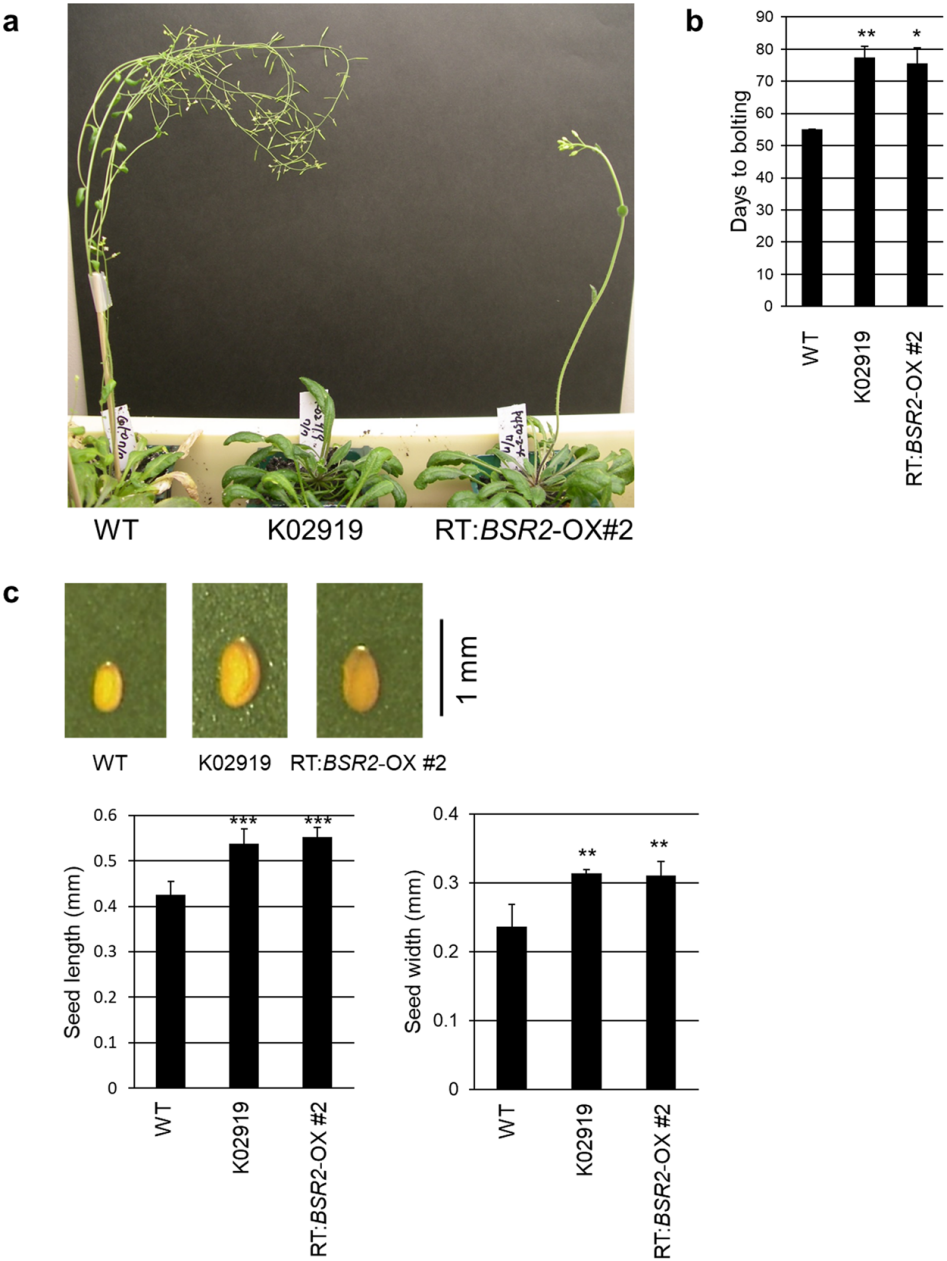
Morphological traits of *BSR2*-OX *Arabidopsis* lines. (**a**) Representative *BSR2*-OX phenotypes 77 d after sowing and (**b**) the number of days to bolting. (**c**) The comparative sizes, lengths and widths of seeds. Asterisks indicate values that are significantly different from the WT (*P < 0.05, **P < 0.01, ***P < 0.001, according to a *t*-test; error bars represent the standard deviation; n = 3 (**b**) and 5 (**c**)).

When *BSR2* was overexpressed in *Arabidopsis*, morphological changes were observed in the leaves, flowers, siliques and seeds, and fertility was remarkably low. The *BSR2-*OX plants grew more slowly than the WT plants (Supplementary Fig. S6a), and they required a longer time to bolt in comparison with WT (Fig. 6a,b). However, the final heights of the *BSR2-*OX plants were taller than those of the WT plants (Supplementary Fig. S6). The flowers, siliques and seeds in the *BSR2*-OX plants were also remarkably larger than those observed in the WT plants (Supplementary Fig. S6 and Fig. 6c).

### Changes in the growth rate and reproductive organ size caused by overexpression or silencing of *BSR2* in rice

**Figure 7:**
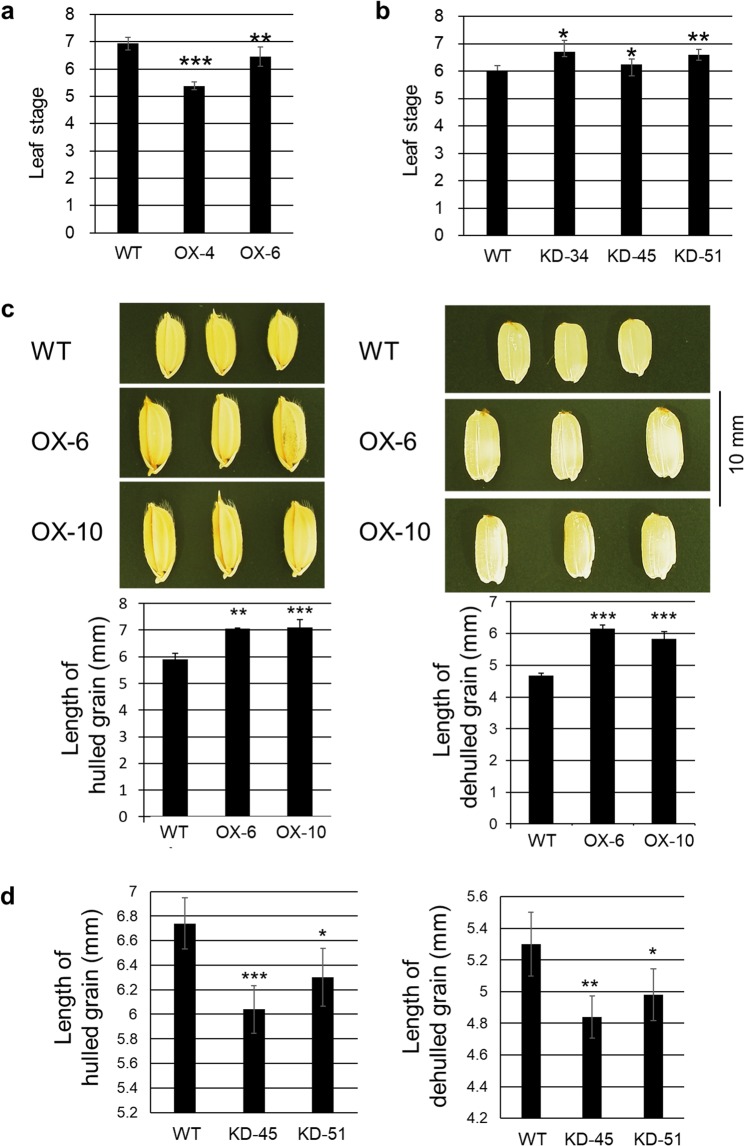
Morphological traits of *BSR2*-OX and -KD rice lines. Leaf stages of (**a**) *BSR2*-OX lines 40 d after sowing and (**b**) *BSR2*-KD lines 32 d after sowing in comparison with WT at the vegetative stage. (**c**) Comparison of hulled grain (left) and dehulled grain (right) of *BSR2*-OX and (**d**) the lengths of hulled grain and dehulled grain of *BSR2*-KD rice lines in comparison with WT. Asterisks indicate values that are significantly different from the WT (*P < 0.05, **P < 0.01, ***P < 0.001, according to a *t*-test; error bars represent the standard deviation; n = 5–12 (**a**), 4–13 (**b**), 4 (**c**) and 5 (**d**)).

When *BSR2* was overexpressed in rice, the *BSR2*-OX plants grew more slowly than the WT plants just like in *Arabidopsis*. The leaf stages of the *BSR2*-OX-4 and -6 lines 40 d after sowing were 5.4 and 6.5, respectively. These values were significantly smaller than that of the WT plants (6.9; Fig. 7a). Further repeats of this experiment showed that the leaf stages of the *BSR2*-OX-6 and -2 plants were also smaller than that of the WT plants (Supplementary Fig. S7). These data indicate that the *BSR2*-OX rice grew more slowly than the WT rice plants. We examined whether the *BSR2-*KD rice had an opposite effect on the leaf growth rate. The leaf stages of three independent *BSR2*-KD lines developed faster than the leaves of the WT plants at the vegetative stage (Fig. 7b), indicating that *BSR2*-KD rice grew faster than WT.

The floral organs in the *BSR2*-OX plants were also larger than those of the WT plants at the flowering stage (19 weeks after sowing). The lemma, glume, anther and pistil lengths of the *BSR2*-OX plants were 13–19%, 25–30%, 29–53% and 22–39% longer than those of WT, respectively (Supplementary Fig. S8). The lengths of unhulled seed and dehulled rice grains from two independent *BSR2*-OX lines at the harvest stage were 20% and 25–32% longer than those of the WT (Fig. 7c). The fertility of the *BSR2*-OX rice plants was also notably low. Conversely, the lengths of unhulled seed and dehulled rice grains from two independent *BSR2*-KD lines were 7–10% and 6–9% shorter than those of the WT (Fig. 7d).

According to the Rice Expression Profile Database^29^ (http://ricexpro.dna.affrc.go.jp/), in WT ‘Nipponbare’ rice, *BSR2* is expressed in reproductive organs such as inflorescences, pistils and lemmas as well as in vegetative organs such as leaves and roots (Supplementary Fig. S9). The spatio-temporal expression profile of *BSR2* does not contradict the morphological phenotype of *BSR2*-OX or -KD rice plants. Therefore, it appears that *BSR2* innately regulates the growth rate and seed size as well as disease resistance in rice.

### Defence-related phytohormone levels in *BSR2*-OX *Arabidopsis*

To examine whether defence-related plant hormones are involved in disease resistance in *BSR2*-OX *Arabidopsis*, the salicylic acid (SA) and jasmonic acid (JA) levels were simultaneously measured from the same plant samples 2 d after *Pst* DC3000 inoculation (Supplementary Fig. S10). The SA levels with or without pathogen infection were similar and the JA levels without pathogen infection were similar between the vector control plants and the *BSR2*-OX plants. Although the JA content of the *BSR2*-OX#2 line after infection appeared to be higher than that of the vector control, the JA content of the *BSR2*-OX#3 line, which has strong resistance to *Pst* DC3000, was similar to that of the vector control. These results suggest that JA and SA are unlikely to be involved in the disease resistance conferred by *BSR2*-OX in *Arabidopsis*.

The ethylene (ET) emission levels were also measured, as described in the Supplementary Methods. The ET emission levels in the *BSR2*-OX and vector control plants were too low to detect even 3 d after *Pst* DC3000 infection. Together, these data show that the SA, JA and ET levels were not significantly different between the *BSR2*-OX plants and the vector control plants in our experiments. Therefore, the disease resistance mechanism associated with BSR2 may be unrelated to the disease resistance mechanisms mediated by these three phytohormones.

## Discussion

### The mechanism of broad-spectrum disease resistance conferred by BSR2

In this study, we describe *BSR2*, a gene that encodes a P450 protein that is involved in innate disease resistance to the necrotrophic fungal pathogen *R. solani*, the causal pathogen of sheath blight in rice. In addition, *BSR2*-OX rice showed resistance to another isolate of *R. solani* that belongs to a different AG and causes brown sheath blight, a different rice disease. In *Arabidopsis*, *BSR2* expression also conferred remarkable resistance to the hemibiotrophic bacterial pathogen *Pst* DC3000, a hemibiotrophic fungal pathogen *C. higginsianum*, and to *R. solani*. To our knowledge, there are few genes that confer such broad-spectrum disease resistance.

To date, several *R. solani* resistance genes have been identified in rice^6–8,10,11^. Some reports suggest the importance of ET/JA signalling pathway in *R. solani* resistance^5,6,8^. Transgenic rice lines with inducible ET production were previously generated by expressing rice *ACS2* (encoding 1-aminocyclopropane-1-carboxylic acid synthase, a key ET biosynthesis enzyme) under the control of a strong pathogen-inducible promoter^6^. These *OsACS2*-overexpression lines had significantly increased levels of endogenous ET and exhibited increased resistance to a field isolate of *R. solani*, as well as to different races of *P. oryzae*. These results suggest that pathogen-inducible ET production in transgenic rice can enhance resistance to necrotrophic *R. solani* as well as to hemibiotrophic *P. oryzae*. ET signalling is also important for isoflavonoid-mediated resistance to *R. solani* in the roots of *Medicago truncatula*^5^. Overexpression of *OsWRKY30* increases endogenous JA accumulation and resistance to *R. solani* and *P. oryzae*^9^. OsWRKY4 protein, which is induced upon *R. solani* infection, specifically binds to the promoter regions of pathogenesis-related (PR) genes. Overexpression of *OsWRKY4* increases the resistance to *R. solani*^10^. Moreover, OsWRKY80, which is induced by *R. solani*, JA and ET, but not by SA, binds to the promoter regions of *OsWRKY4*. Similar to *OsWRKY4*, overexpression of *OsWRKY80* increases resistance to *R. solani*, while suppression of *OsWRKY80* by RNAi reduces resistance to *R. solani*. These studies suggest that the OsWRKY80-OsWRKY4 module functions as a positive regulatory circuit for rice defence against *R. solani* that acts via the JA/ET-dependent signalling pathway^8^.

Other reports describe the contribution of SA-dependent immunity to resistance against *R. solani*^30,31^. Kouzai *et al*.^31^ recently found that foliar pretreatment with SA can induce *R. solani* sheath blight resistance in rice and *Brachypodium distachyon*. They also showed the involvement of SA in *R. solani* resistance using SA-deficient *NahG* transgenic rice. Because SA-dependent plant immunity generally targets biotrophs and hemi-biotrophs, their findings suggest a preceding biotrophic stage and a subsequent necrotrophic stage in the *R. solani* infection process^31^. Moreover, overexpression of *Arabidopsis NPR1*, which is responsible for SA signalling, confers resistance to *R. solani* in rice^30^. NPR1 may also target the biotrophic stage of *R. solani*.

The disease resistance mechanism of *BSR2* is still unknown. Because *BSR2* encodes a P450 enzyme, it should be involved in the biosynthesis of certain chemical compounds. Although several reports implicate known defence hormones, the measurement of phytohormones in the present study suggested that *BSR2* overexpression does not affect the production of SA, JA or ET (Supplementary Fig. S10). Some P450 proteins are responsible for the biosynthesis of phytoalexins; for example, PAD3 (CYP71B15) for camalexin^32^, CYP76M7 for phytocassane^33^ and CYP82C2 for 4-hydroxyindole-3-carbonyl nitrile (4-OH-ICN)^34^. However, there are no known chemicals, including plant hormones, that can explain the concomitant phenotypes of seed size and growth rate in addition to the broad-spectrum disease resistance in *BSR2*-OX plants. Therefore, we think that BSR2 may be involved in the biosynthesis of novel chemical compounds that function pleiotropically in disease resistance, seed size and the plant growth rate.

### Positive effect of BSR2 on the reproductive organ size

Seeds of *BSR2*-OX *Arabidopsis* and -OX rice are longer than WT seeds and those of *BSR2*-KD rice are shorter, which suggests that BSR2 is involved in the development of reproductive organs (Figs 6 and 7, Supplementary Figs S6 and S8). There are many reports on the morphological effects of the CYP78A family proteins. KLUH(KLU)/CYP78A5 was the first member of the CYP78A family to be identified in *Arabidopsis*. Whereas CYP78A5 loss-of-function mutants form smaller organs than WT, CYP78A5 overexpression leads to larger organs with more cells. Therefore, it seems that *CYP78A5* controls the sizes of plant organs such as leaves, sepals, petals, ovules and seeds, by promoting cell proliferation^35–37^.

*Arabidopsis* CYP78A6, A8 and A9 are homologous with BSR2 (Supplementary Fig. S2). Overexpression of *CYP78A9* induces longer sepals, petals and pistils, shorter stamens, larger fruit and seed, and reduced fertility^38,39^. The *cyp78a8* and *cyp78a9* double mutant has reduced seed set due to development arrest of the outer ovule integument that leads to female sterility^39^. Similarly, overexpression of *EOD/CYP78A6* increases seed size by promoting both cell proliferation and cell expansion, whereas knockout mutants form small seeds. *CYP78A6* functions redundantly with *CYP78A9* to affect seed growth^40^. Together, these studies show that CYP78A5, A6, A8 and A9 also have positive effects on plant reproductive organ size.

In the monocot rice, *GIANT EMBRYO* (*GE*) is essential for controlling the size balance between embryo and endosperm, and has been reported as encoding CYP78A13 (Supplementary Fig. S2)^41,42^. In contrast to the loss-of-function mutant that had large embryos and small endosperms, *GE* overexpression led to the production of small embryos and enlarged endosperms^41,42^. Moreover, *GE* overexpression promoted cell proliferation and cell expansion that resulted in longer leaves and bigger spikelet hulls^42^. When *CYP78A10*, an *Arabidopsis* ortholog for *GE*, was overexpressed in *Arabidopsis*, the seeds were much larger than those of the WT plants^42^. Furthermore, *CYP78A13* rescues the phenotype of short sepals and siliques in a *CYP78A5* mutant, *Arabidopsis klu-4*, suggesting a conserved role in reproductive organ development in both monocots and dicots^43^. In the dicot tomato, SIKLUH regulates fruit size, which is a domestication trait^44^. In soybean and wheat, GmCYP78A10, GmCYP78A72 (homologous to CYP78A5) and TaCYP78A5 regulate seed size^45–47^. Together, these data suggest that the positive effects of CYP78As on the sizes of the reproductive organs are likely to be conserved in angiosperms. Therefore, the positive effect of BSR2 on reproductive organ size seems to be a typical effect of CYP78A proteins acting to increase cell proliferation and/or expansion. However, in contrast to GE/CYP78A13, *BSR2* overexpression has little effect on the size balance between the embryo and the endosperm (Fig. 7c, right).

### Negative effect of BSR2 on the growth rate

*BSR2*-OX *Arabidopsis* and *BSR2*-OX rice showed slow growth, while *BSR2*-KD rice displayed fast growth (Fig. 7, Supplementary Figs S6 and S7). A *PLA1*/*CYP78A11* loss-of-function mutant in rice has rapid leaf initiation and a shortened plastochron, the time interval during which new leaves are produced^48^. In contrast, transgenic maize constitutively overexpressing maize *PLA1* grow more slowly^49^. A *KLU/CYP78A5* loss-of-function mutant in *Arabidopsis* also showed an accelerated leaf initiation rate, suggesting that *KLU/CYP78A5* affects the plastochron as well as the organ size^50^. The shortened plastochron duration was mostly due to the increased cell division rate. Wang *et al*.^50^ also suggested the possibility that the plastochron duration and organ size are coordinately regulated by an unknown common regulator. Due to its sequence similarity at the protein level, the BSR2 protein may produce chemicals similar to the PLA1 or KLU proteins. Such chemicals may be responsible for the repression of leaf initiation observed and allow for the maintenance of an appropriate plastochron duration. *BSR2* overexpression could increase the production of such chemicals. This might suppress the rate of leaf initiation by decreasing the rate of cell division, leading to a longer plastochron and slower growth.

### Possible function of BSR2

Although some members of the CYP78As, such as CYP78A5, are known to affect seed size and plastochron, no member of the CYP78As has been reported as being involved in disease resistance. *BSR2*-KD rice displayed susceptibility to *R. solani* and had shorter seeds and faster growth than WT plants, probably due to a shorter plastochron. These data suggest that *BSR2* is involved in pleiotropic functions such as innate immunity, reproductive organ size and growth rate in rice.

Our discovery and characterization of *BSR2* adds new insight into the biological roles of CYP78As, because, to our knowledge, no other CYP78A has such pleiotropic function. CYP78As are found in most angiosperm species and moss, suggesting that they have an ancient conserved role^28^. This is the first report on disease resistance found by overexpression of a *CYP78A* (*BSR2*), and it is quite possible that other members of the CYP78A family play key roles in disease resistance as well.

The biochemical function of BSR2 is unknown. *Zea mays* CYP78A1 has been reported as having lauric acid 12-monooxygenase activity^51^. Similarly, CYP78A5, A7 and A10 have short-chain fatty acid hydroxylase activities^52^. CYP78A5, A6, A9 and A11 act non-cell-autonomously; therefore, they are also thought to be involved in generating a novel mobile growth signal that is distinct from the classical phytohormones^38,40,48^. Analyses of gain-of-function and loss-of-function mutants of *CYP78A9* (one of the closest homologues of *BSR2*) have revealed perturbations in the flavonol biosynthesis pathway^39^. The same study found no clear overlap between the *CYP78A9*-regulated genes and known phytohormone-responsive genes^39^. Moreover, an earlier study found that no known phytohormone treatment could mimic the entire phenotype of *CYP78A9*-OX *Arabidopsis*^38^. No known phytohormone could also mimic the pleiotropic phenotype of *BSR2*-OX plants. Because CYP78As are involved in fatty acid and flavonol biosynthesis, it is conceivable that *BSR2* overexpression led to the activation of a novel biosynthetic pathway. Such a pathway could have several bioactive products that affect the phenotypes associated with BSR2 expression; including reproductive organ size, growth rate and disease resistance. In future, the biochemical function of BSR2 should be characterized. RNA sequencing analysis coupled with a metabolomic assay would shed light on which genes or gene products are activated by *BSR2* overexpression during the vegetative and reproductive stages and before and after infection by *R. solani*.

### Applications for disease resistant crops

*BSR2* may be useful for generating crop plants that have broad-spectrum disease resistance. Because *BSR2* was effectively expressed in both the monocot rice and the dicot *Arabidopsis*, it may be useful for generating many monocot and dicot crops. However, because *BSR2* overexpression led to notably reduced fertility in both *Arabidopsis* and rice, it would be useful to use promoters that prevent *BSR2* expression in the reproductive organs. Alternatively, the adoption of pathogen-inducible promoters like *PR1b*^53^ may be useful for preventing the growth retardation and low fertility that result from constitutive expression of *BSR2*. However, constitutive *BSR2* expression may still be applicable to crops that are generally propagated without reproductive organ development; for example, potato, sweet potato and sugarcane. In *Arabidopsis*, *BSR2* overexpression also resulted in enlarged siliques (fruit) and flowers. Therefore, *BSR2* expression may be beneficial in ornamental flower and fruit tree species, in addition to the broad-spectrum disease resistance that it confers.

## Methods

### Plant materials and culture conditions

*Arabidopsis thaliana* ecotype Columbia (Col-0) was used as the WT control. A mutant line, *cpr5-2* (a gift from Dr. B.N. Kunkel, Washington University, USA), with very high resistance to *Pst* DC3000 was used as positive control. The *Arabidopsis* ecotype Eil-0, which is highly resistant to *C. higginsianum*, was also used as positive control. The candidate full-length rice cDNAs identified by *Pst* DC3000 screening were re-transformed in *Arabidopsis* as previously described^23^. Transgenic *Arabidopsis* plants were grown on half-strength MS medium (Wako Pure Chemicals, Osaka, Japan) with 1% sugar, B5 vitamins (0.04% myo-inositol, 0.0004% nicotinic acid, 0.0004% pyridoxine hydrochloride, 0.004% vitamin B1 hydrochloride), 0.05% MES, 10 µg/mL hygromycin B (Wako Pure Chemicals) and 0.8% agar adjusted to pH 5.7. Col-0, *cpr5-2* and Eil-0 seeds were sown in the same medium but without the hygromycin. For the *R. solani* resistance assay, 2-week-old plants were transferred to pots containing sterile moistened black peat moss (Super Mix; Sakata, Yokohama, Japan) and grown under short-day conditions (9 h light and 15 h dark) at 22 °C. To observe morphological traits or harvest seeds, 3- to 4-week-old plants were transferred to pots and grown under long-day conditions (16 h light and 8 h dark) at 22 °C.

Rice (*Oryza sativa* L.) cultivar ‘Nipponbare’ was used as the WT control. Dehusked seeds were surface sterilized and sown on half-strength MS medium containing 3% (w/v) sucrose and 0.4% (w/v) Gelrite (Wako Pure Chemicals) in sterile Petri dishes. The dishes were incubated in a growth chamber at 28 °C in the dark for 3 d, followed by 4–7 d at 25 °C under long-day conditions (16 h light [60–70 µmol m ^−2^ s ^−1^] and 8 h dark). For transgenic selection, hygromycin B (30–50 µg/mL) was added to the medium. WT seedlings and hygromycin-resistant transgenic seedlings were transplanted to pots containing soil (Bonsol No. 2, Sumitomo Kagaku Kougyo, Osaka, Japan) and cultivated in a greenhouse at 27–30 °C.

### Pathogen strains and cultures

The bacterial pathogen *Pst* DC3000 and the fungal pathogen *C. higginsianum* (MAFF305635) were used for disease resistance screening in *Arabidopsis*. The fungal pathogen *R. solani* (MAFF243956) was used for both disease resistance screening in *Arabidopsis* and evaluation of sheath blight disease resistance in rice. In addition, the *R. solani* Shimomura 9 isolate was kindly provided by Dr. Toshiyuki Morikawa (Toyama Prefectural Agricultural, Forestry and Fisheries Research Center) and used to evaluate resistance to brown sheath blight in rice.

The protocols for *Pst* DC3000 culture and inoculation are as previously described^23^. The fungi *C. higginsianum* and *R. solani* were cultured on PDA agar plates (0.39% potato extract, 2.1% glucose and 1.41% agar, adjusted to pH 5.6; Nissui, Tokyo, Japan) at 28 °C under dark conditions for 2 weeks and 3 d, respectively, before inoculation.

### *Pst* DC3000 and *C. higginsianum* screening

*Arabidopsis* Col-0 was used as the WT (negative control), and *cpr5-2* (a *Pst* DC3000 resistant mutant line) and Eil-0 (a *C. higginsianum* resistant ecotype) were used as positive controls. The control ecotype, *cpr5-2* and an additional 1,000 rice-FOX *Arabidopsis* lines^16,23^, which were different from those screened in the previous report^23^, were sown at 5 seeds per well in 60-well plates containing pre-sterilized moist black peat moss. Screening for *Pst* DC3000 and *C. higginsianum*, and the bacterial growth assay for *Pst* DC3000 were performed as previously described^23^.

### Quantification of relative fungal growth by quantitative real-time PCR

Quantification of *C. higginsianum* growth was performed as follows. Four- to five-week-old *Arabidopsis* plants were inoculated by spraying the rosette leaves with *C. higginsianum* (3.2 × 10^6^ conidia/mL). *C. higginsianum* infection was quantified by measuring *C. higginsianum* genomic DNA relative to *Arabidopsis* genomic DNA using quantitative real-time PCR (qRT-PCR) analysis. Total DNA was extracted from the aerial parts of the plants using extraction buffer containing 0.5% (w/v) sodium dodecyl sulfate, 250 mM EDTA, 25 mM NaCl, and 200 mM Tris-HCl (pH 8.0), as previously described^54^. Disease severity was evaluated by comparing the amounts of *C. higginsianum* genomic *ACT* DNA relative to *Arabidopsis* genomic *ACT2* (At3g18780) DNA 3 d after inoculation.

Quantification of *R. solani* in rice was performed as follows. Total DNA was extracted from a previously inoculated leaf, excluding 5-mm portions on either side of the inoculation point. The disease severity was evaluated by the amounts of *R. solani* ITS3 of rDNA relative to rice *Rubq1* (Os06g0681400) using qRT-PCR. The same primers were used for two different *R. solani* strains. The primer sequences are listed in Supplementary Table S2.

### Assay for resistance to *R. solani* in *Arabidopsis*

*Arabidopsis* plants were grown under aseptic short-day conditions for 6 weeks. Three-day-old *R. solani* (MAFF243956) on PDA medium (50 mL) was crushed using a mortar and pestle and mixed with an equal volume of sterile water (50 mL). A drop (5 μL) of the mycelial agar liquid suspension was applied to each of the middle rosette leaves of the *Arabidopsis* plants. The plants were then incubated under humid short-day conditions at 22 °C and the disease lesion length was measured one week after inoculation.

### Plasmid construction and rice transformation

For the *BSR2* overexpression lines, the full-length AK072163 cDNA (kindly provided by the Rice Genome Resource Center, NARO, Japan) was inserted into the *Sfi*I site of the pRiceFox vector^55^ between the maize (*Zea mays*) *ubiquitin* promoter and the *nopaline synthase* terminator. For the *BSR2* knockdown lines, a PCR product that was designed to trigger RNAi was first subcloned into the pENTR/D-TOPO entry vector (Invitrogen). The PCR product was then amplified using the following primers: 5′-GTGGGACTAAGACGAGGAGA-3′ and 5′-GAGCTATTCTACACTCATCA-3′. The fragment was used to make an inverted repeat construct in the pANDA destination vector through a LR clonase reaction by the Gateway system^56,57^. To generate overexpression and knockdown rice plants, the constructed vectors were transformed into rice using *Agrobacterium*-mediated method^58^. The T0 transformant plants and their progeny were used for morphological observations and to test *R. solani* resistance.

### Detached leaf inoculation assay for *R. solani* in rice

The detached leaf inoculation assay using *R. solani* was performed as follows. Leaf blades were detached from rice plants at the tillering or heading stages. Three-day-old *R. solani* (MAFF243956 or Shimomura 9 isolate) growing on PDA medium (50 mL) was crushed using a mortar and pestle and mixed with sterile water (25 or 50 mL). Detached leaves were placed on a wet paper towel in Petri dishes, and 30 μL of the mycelial agar liquid suspension was drop-inoculated onto the middle parts of the leaves. Inoculated leaves were incubated in humid long-day conditions at 25 °C and after 7–13 d disease development was evaluated either by measuring the lesion length or by quantifying the relative fungal growth using qRT-PCR.

### RNA extraction and quantitative real-time RT-PCR analysis

Total RNA was extracted and purified from rice leaves as described previously^25^, or by using Sepasol-RNA Super G reagent (Nacalai Tesque, Kyoto, Japan) according to the manufacturers protocol. Total RNA was extracted from *Arabidopsis* leaves using an RNeasy mini kit (Qiagen, Valencia, CA, USA). First-strand cDNAs were synthesized from equal amounts of total RNA in 10 or 20 μL using a PrimeScript II first-strand cDNA synthesis kit (Takara, Tokyo, Japan) or a ReverTra Ace qPCR RT Master Mix with gDNA Remover Kit (Toyobo, Osaka, Japan), according to the manufacturers’ protocols. Thermal Cycler Dice TP800 system (Takara) and a Kapa SYBR FAST qPCR kit (Kapa Biosystems, Cape Town, South Africa) were used for the qRT-PCR, as described by the manufacturer. The list of primers used for qRT-PCR are included in Supplementary Table S2. The transcript levels of each gene were normalized to an endogenous rice reference gene, *Rubq1*^59^. *Actin2* was used as the reference gene in *Arabidopsis* and for quantifying the relative fungal growth (Supplementary Table S2). The relative expression level of each gene was calculated using the 2^−ΔΔCt^ expression ratio, which corrects for gene-specific PCR amplification efficiencies^60^.

### Sequence alignment and phylogenetic analysis

The sequence alignment and phylogenetic analysis were performed as described in Supplementary Methods.

### *R. solani s*heath inoculation assay in rice

The *R. solani* sheath inoculation assay was performed in rice as described in Supplementary Methods.

### Phytohormone measurements

Phytohormones were measured as described in Supplementary Methods.

## Supplementary information

Supplementary Information

## Data Availability

All data generated or analysed during this study are included in this article and its Supplementary Information files.
