# Supplementary material for: The rice CYP78A gene BSR2 confers resistance to Rhizoctonia solani and affects seed size and growth in Arabidopsis and rice
Source: Sci Rep. 2019 Jan 24;9:587. doi: 10.1038/s41598-018-37365-1 (PMC6345848; doi:10.1038/s41598-018-37365-1)
Supplement: Supplementary file 1 — Supplementary Information [file 41598_2018_37365_MOESM1_ESM.pdf]

The rice *CYP78A* gene *BSR2* confers resistance to *Rhizoctonia solani* and affects seed size and growth in *Arabidopsis* and rice

Satoru Maeda[1], Joseph G. Dubouzet[1], Youichi Kondou[2], Yusuke Jikumaru[2], Shigemi Seo[1], Kenji Oda[3], Minami Matsui[2], Hirohiko Hirochika[1] and Masaki Mori[1,*]

[1]Institute of Agrobiological Sciences, NARO (NIAS), Tsukuba, Japan

[2]RIKEN Yokohama, Tsurumi, Yokohama, Japan

[3]Research Institute for Biological Sciences, Okayama Prefectural Technology Center for Agriculture, Forestry and Fisheries, Okayama, Japan

[*]Correspondence and requests for materials should be addressed to M.M.o. (e-mail: morimasa@affrc.go.jp)

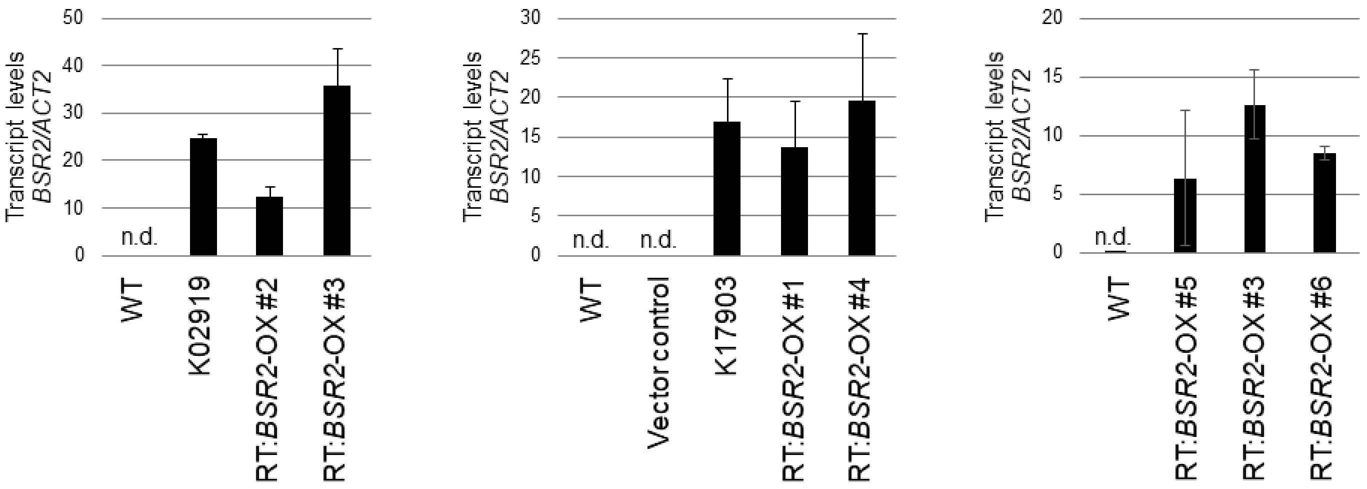

Supplementary Figure S1. Transcript levels of *BSR2* in *BSR2*-OX *Arabidopsi*s lines. n.d.; not detected. Error bars represent the standard deviation; n = 3–5.

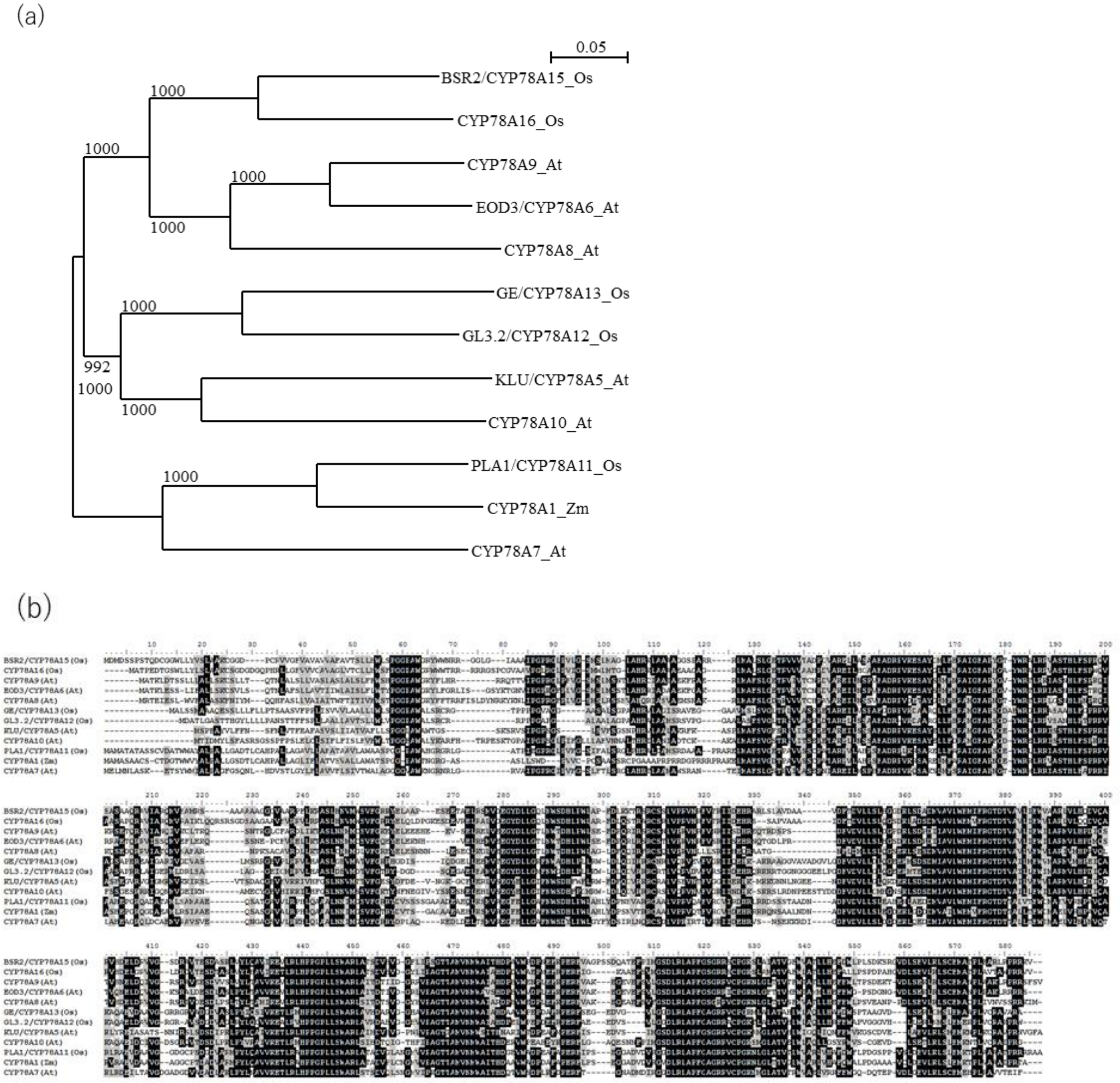

Supplementary Figure S2. Protein sequence analyses between BSR2 and its related cytochrome P450s. Phylogenetic tree (a) and alignment (b) for BSR2 and its related P450s (see Supplementary Methods). Protein sequence data are from rice (BSR2/CYP78A15/Os08g0547300, CYP78A16/Os09g0528700, GE/CYP78A13/Os07g0603700, GL3.2/CYP78A12/Os03g0417700 and PLA1/CYP78A11/Os10g0403000), *Arabidopsis* (KLU/CYP78A5/AT1G13710, EOD3/CYP78A6/AT2G46660, CYP78A7/AT5G09970, CYP78A8/AT1G01190, CYP78A9/AT3G61880 and CYP78A10/AT1G74110) and maize (CYP78A1/P48420). At, *Arabidopsis thaliana*; Os, *Oryza sativa*; Zm, *Zea mays*. Numbers at nodes indicate bootstrap values. The bar corresponds to 0.05 amino acid substitutions per site. Black and gray backgrounds indicate identical and similar amino acids, respectively.

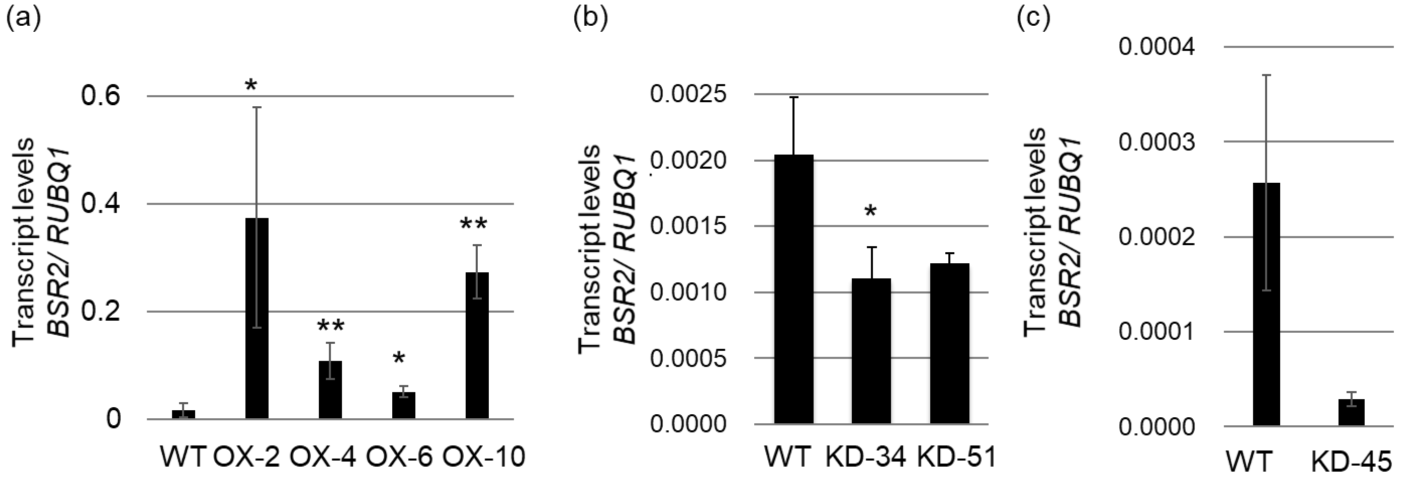

Supplementary Figure S3. Transcript levels of *BSR2* in *BSR2*-OX (a) and -KD (b and c) rice lines. Asterisks indicate that values are significantly different from the WT (*P<0.05, **P<0.01 according to a *t*-test; error bars represent the standard deviation; n = 3–4(a), 3–4 (b) and 3 (c)).

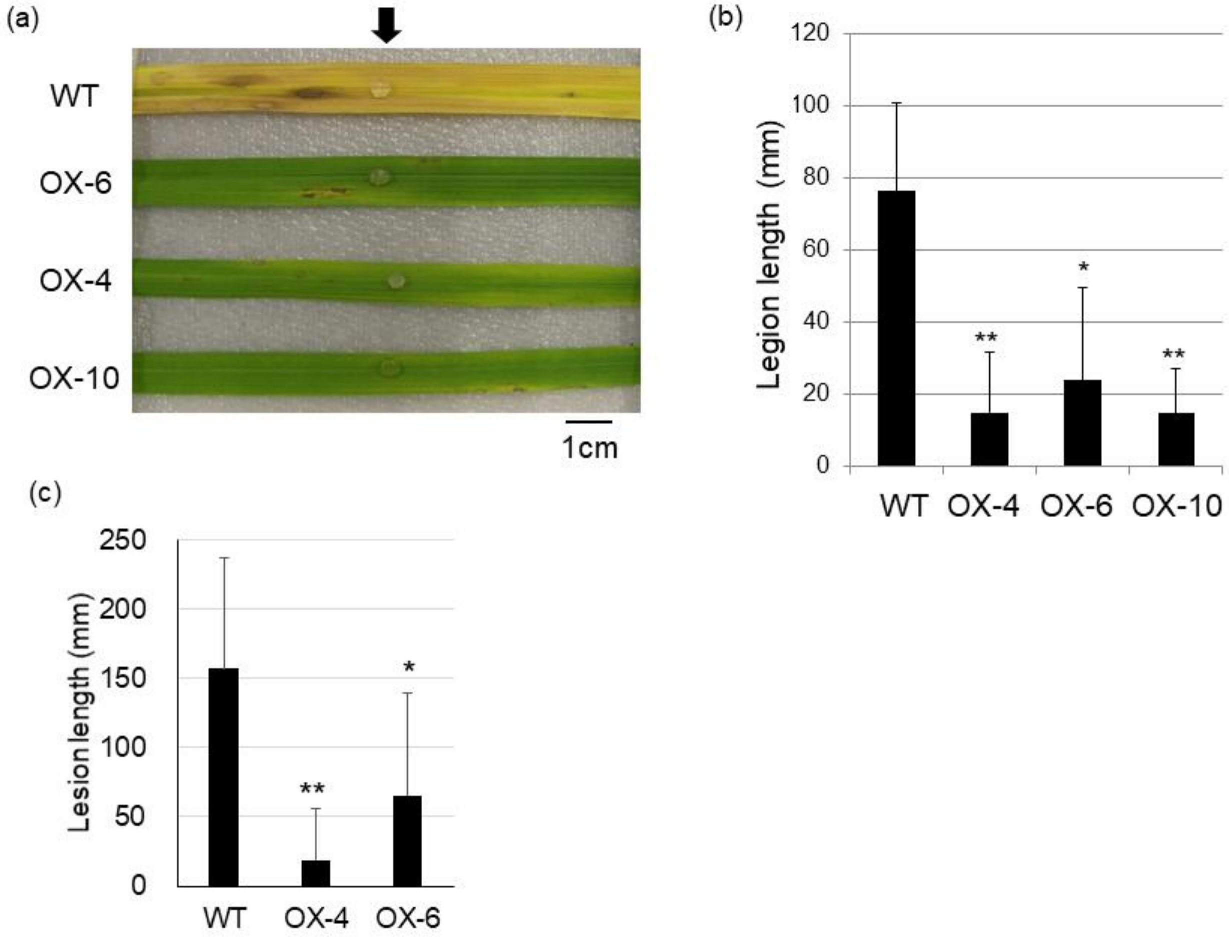

Supplementary Figure S4. Repeated experiment by detached leaf inoculation assay and sheath inoculation assay for sheath blight resistance in *BSR2*-OX rice lines.

Comparisons of (a) inoculated leaves and (b) lesion lengths of detached leaf blades from *BSR2*-OX lines 7 d after drop inoculation with *R. solani* (MAFF243956; AG1-1A). The third leaf blades from the flag leaf at heading stage were used for inoculation. The inoculation points are indicated by an arrow. (c) Result of sheath inoculation assay using rice plants (see Supplementary Methods). Comparisons of lesion lengths of flag leaves 2 weeks after sheath inoculation. Asterisks indicate that values are significantly different from the WT (*P<0.05, **P<0.01 according to a *t*-test; error bars represent the standard deviation; n = 4 (b) and 4–8 (c)).

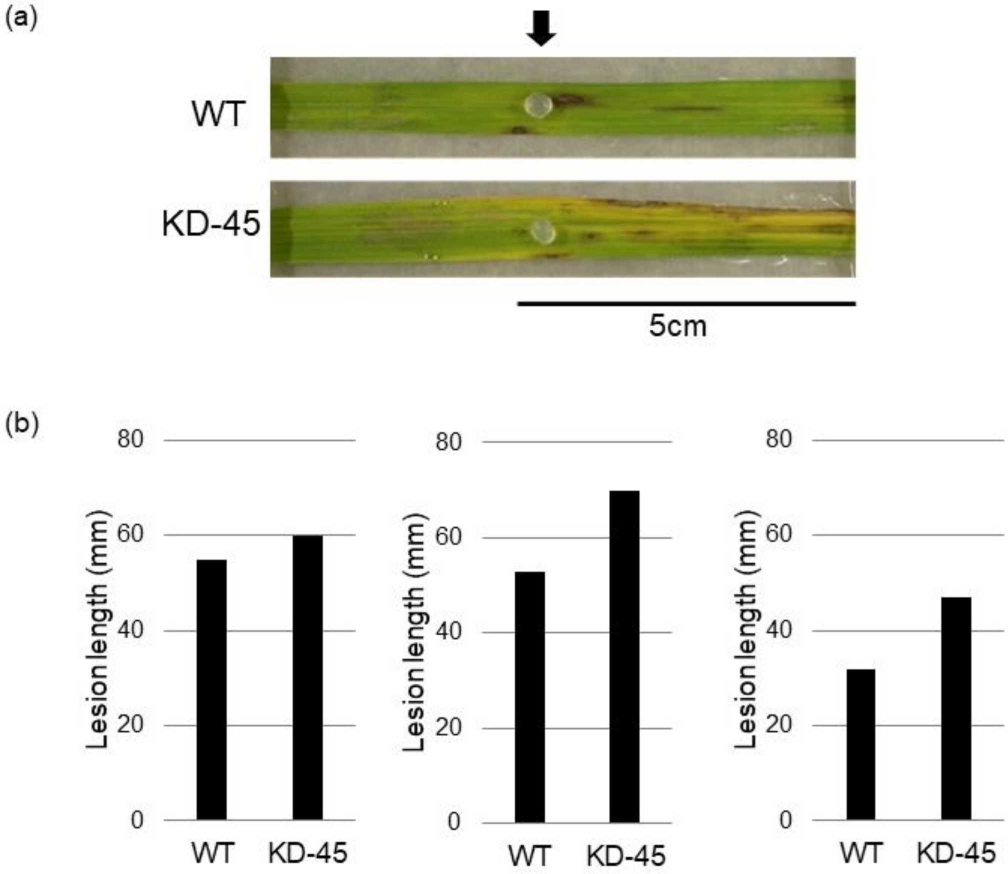

Supplementary Figure S5. Resistance to *R. solani* (AG-1 IA) sheath blight in a *BSR2*-KD rice line.

Comparison of (a) inoculated leaves and (b) lesion lengths of detached leaf blades of *BSR2*-KD lines 8 d after drop inoculation. The second leaf blades from the top leaf at leaf stage 11 were used for inoculation. The lesion lengths in the *BSR2*-KD plants were longer than those in the WT plants. Tests were performed three times with similar results. The inoculation points are indicated by an arrow.

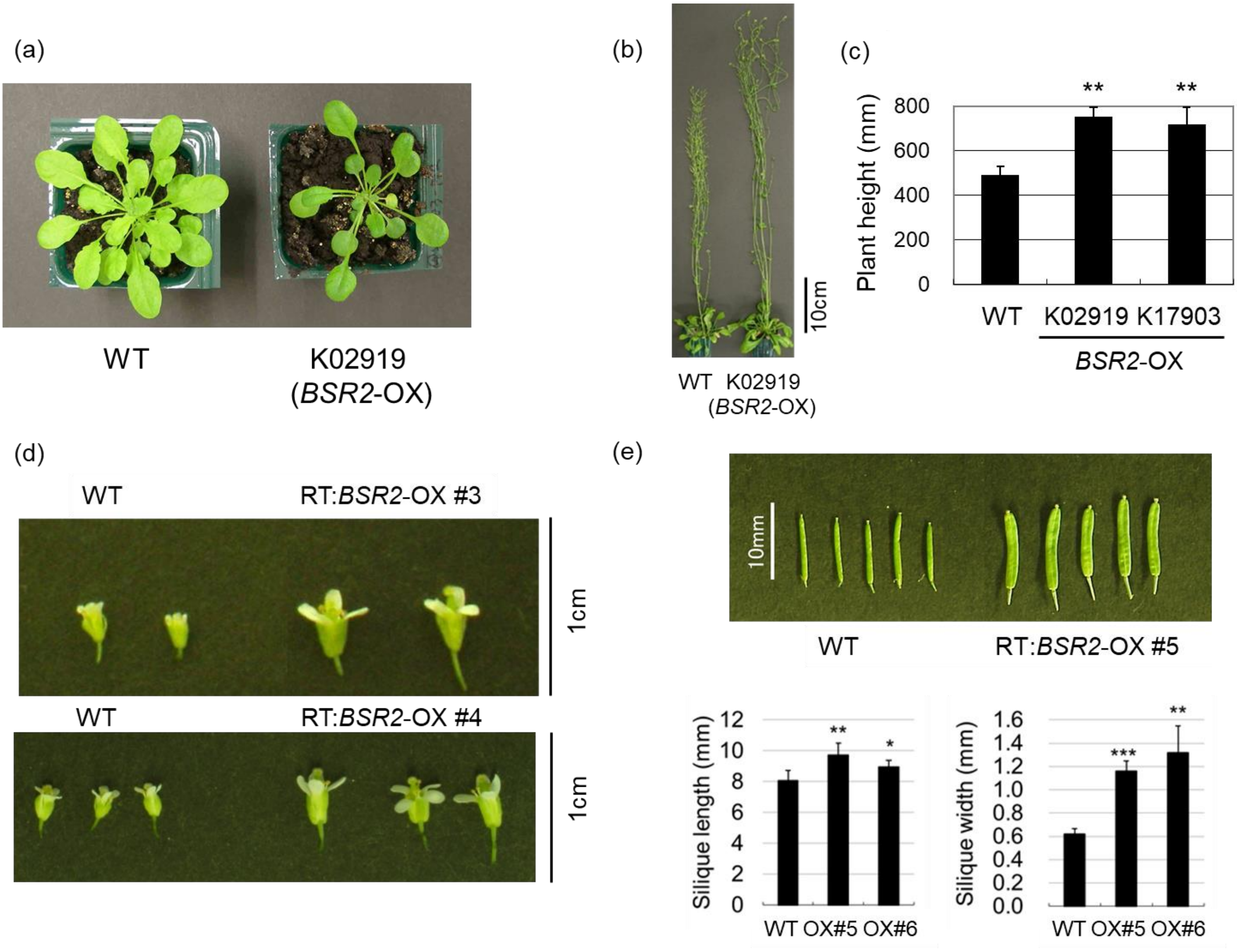

Supplementary Figure S6. Morphological traits of *BSR2*-OX *Arabidopsis* lines.
Adult plants at the vegetative stage (a) and the reproductive stage (b). (c) Plant heights at the harvest stage. (d) Comparison of flowers. (e) Comparison of siliques, lengths and widths of siliques. OX#5, RT:*BSR2*-OX #5; OX#6, RT:*BSR2*-OX #6. Asterisks indicate that values are significantly different from the WT (*P<0.05, **P<0.01, ***P<0.001, according to a *t*-test; error bars represent the standard deviation; n = 5–10 (c), 5 (e)).

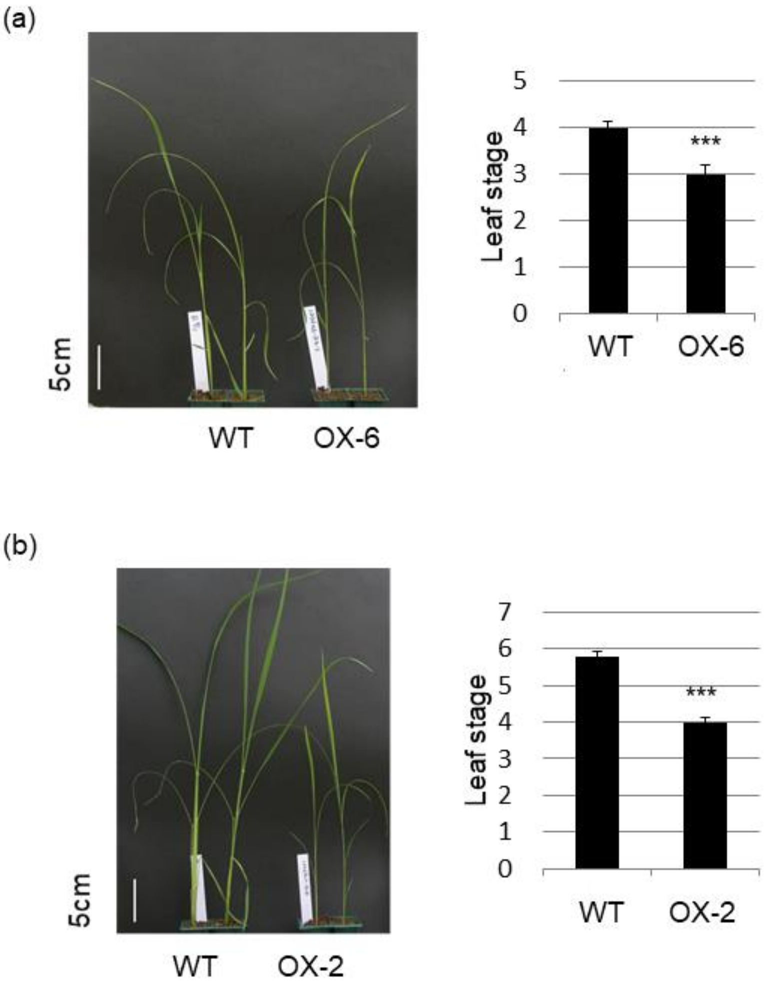

Supplementary Figure S7. Leaf stages of *BSR2*-OX rice lines at the vegetative stage.
Leaf stages of the OX-6 line 24 d after sowing (a) and the OX-2 line 35 d after sowing (b). Asterisks indicate that values are significantly different from the WT (***P<0.001, according to a *t*-test; error bars represent the standard deviation; n = 4–8).

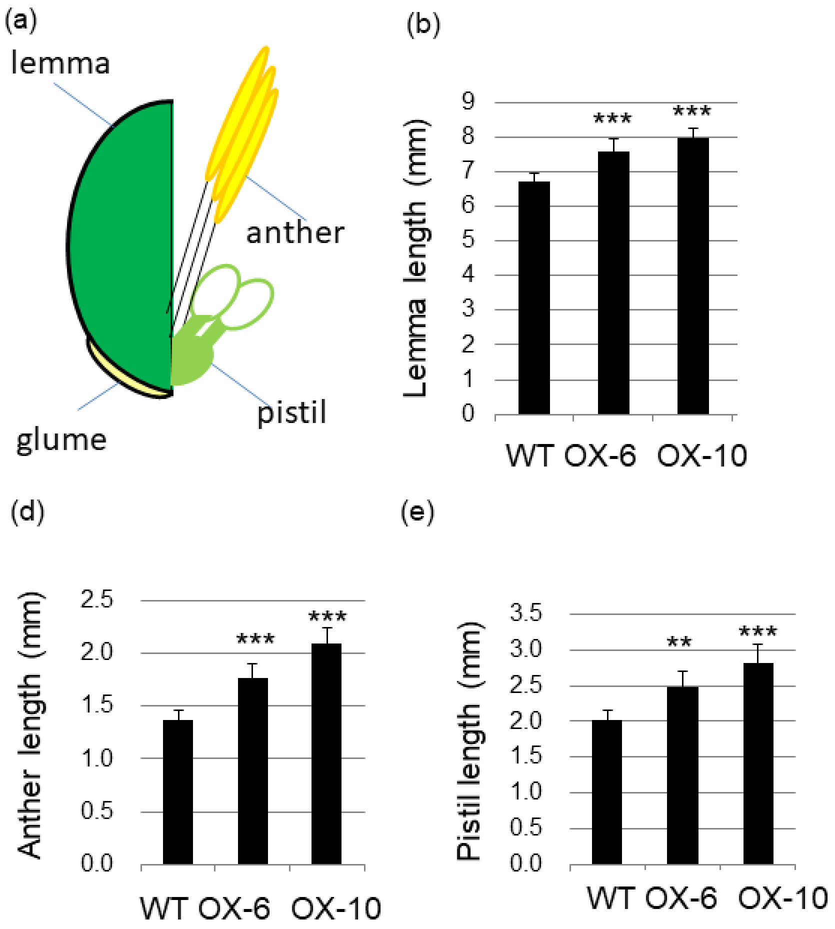

Supplementary Figure S8. Sizes of floral organs in the flowering stage in *BSR2*-OX rice lines.

(a) Schematic representation of rice flower. The comparative lengths of seeds (b), glumes (c), anthers (d) and pistils (e). Asterisks indicate that values are significantly different from the WT (**P<0.01, ***P<0.001, according to a *t*-test; error bars represent the standard deviation; n = 6).

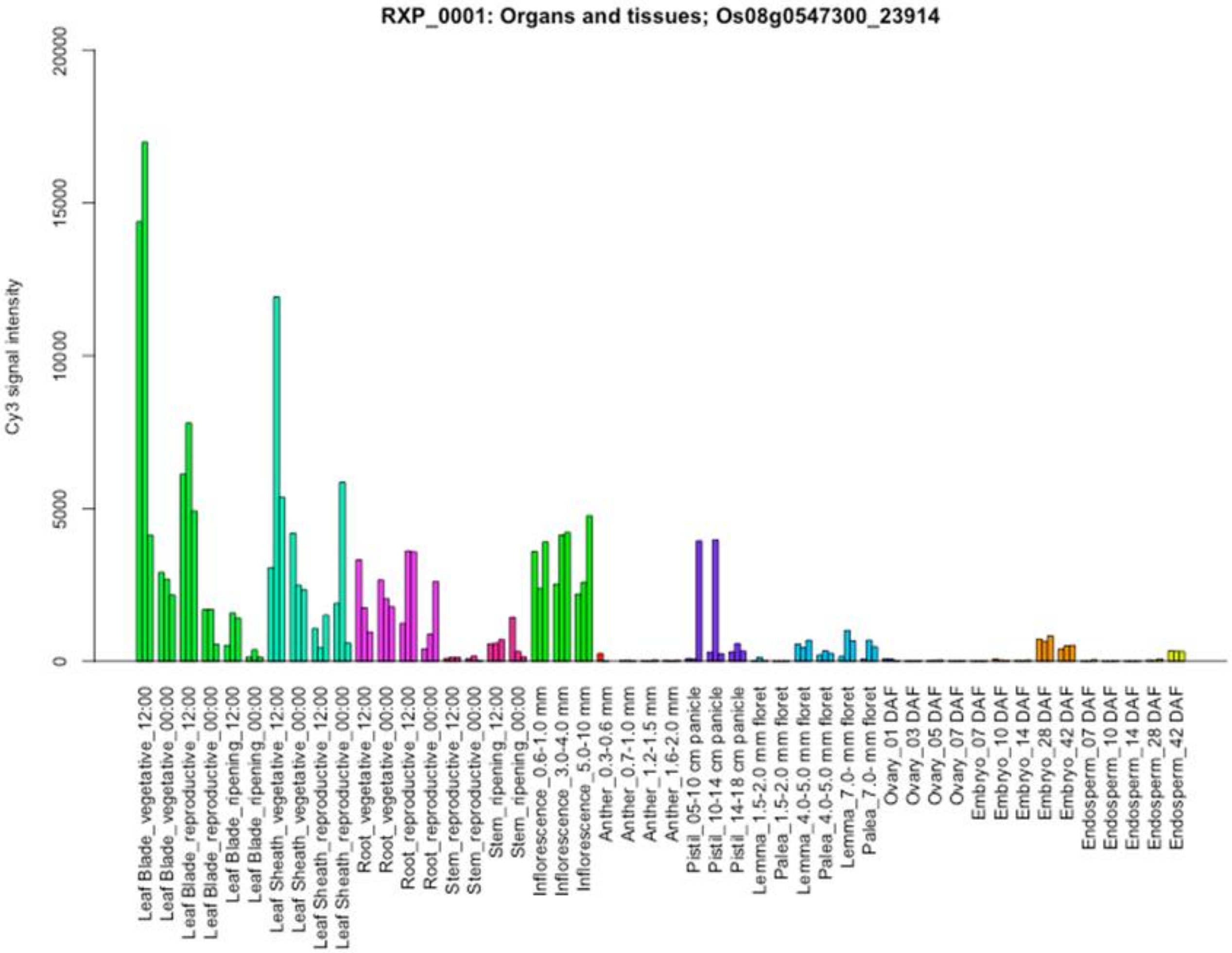

Supplementary Figure S9. Organ and tissue specificity of *BSR2* expression in rice by Rice Expression Profile Database.

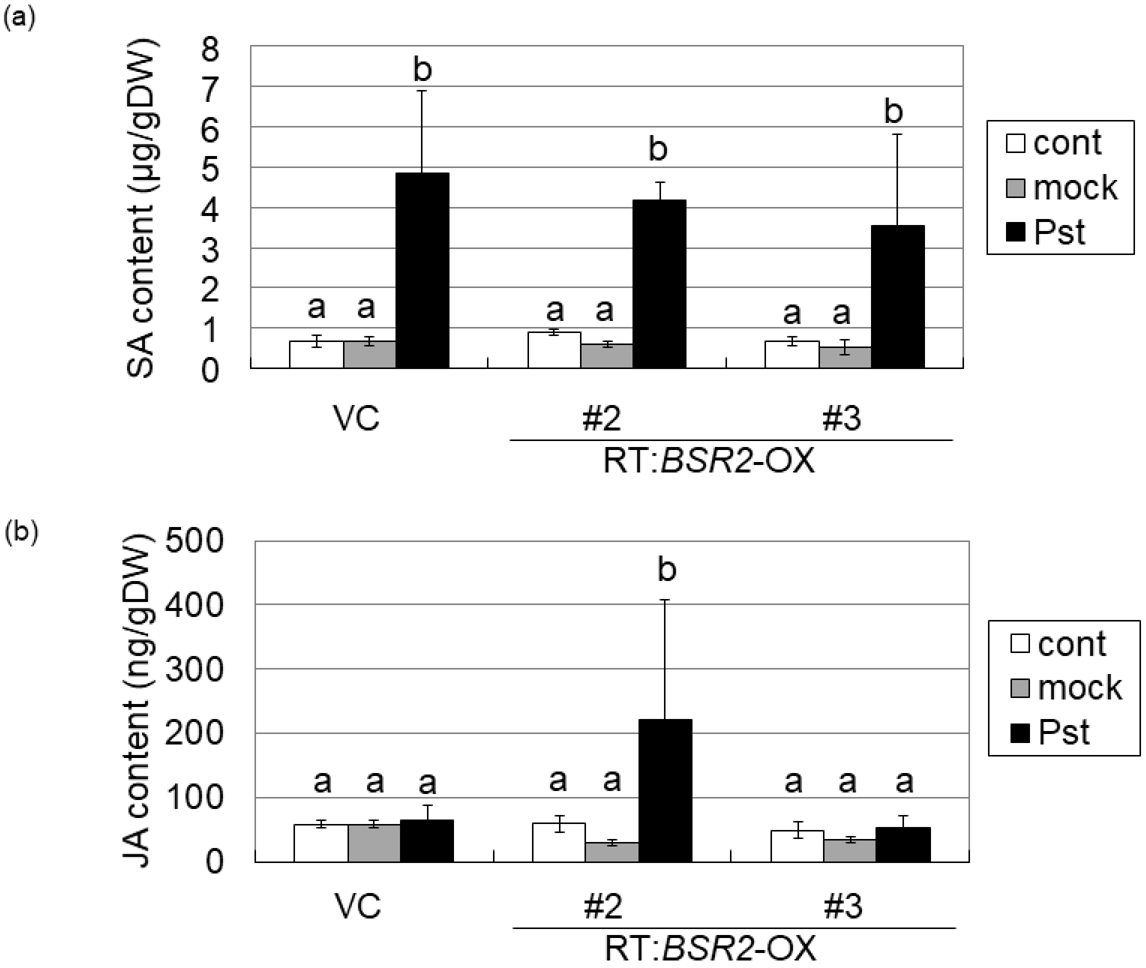

Supplementary Figure S10. Hormone contents of *BSR2*-OX *Arabidopsis* lines.
Salicylic acid (a) and jasmonic acid (b) contents in leaves were measured with or without pathogen infection (see Supplementary Methods). SA, salicylic acid; JA, jasmonic acid; DW, dry weight; VC, vector control; cont, control; Pst, *Pseudomonas syringae* pv. *tomato* DC3000. Different letters indicate significant differences (*$P<0.05$, according to a Tukey’s-test; error bars represent the standard deviation; n =4).

Supplementary Table S1. Rice full-length cDNA inserts that generated resistance to both *Pst* DC3000 and *C. higginsianum* in *Arabidopsis* and their performance against *R. solani*.

Line No.[1]	Original line	Independently transformed line(s)[2]	Accession No.	RAP ID[3]	RAP description[3]	Response to *R. solani*[4]
Resistance confirmed by independent line(s) (7 lines)						
0	K02919	K17903, RT	AK072163	Os08t0547300-01	Similar to cytochrome P450 78A1. (*BSR2*, this work)	R
2	K21617	RT	AK103699	Os10t0530900-01	Similar to glutathione S-transferase GST 30.	S
3	K00841	RT	AK072201	Os01t0503400-04	Similar to metal transporter Nramp6.	
4	K15424	R06015, RT	AK070024	Os09t0533600-01	Similar to Avr9/Cf-9 induced kinase 1. (*BSR1*)	S
6	K25904	K18218(2 inserts)	AK072899	Os09t0363900-01	Similar to HOTHEAD protein precursor.	
7	K02342	K23019	AK102525	Os12t0619000-01	IQ calmodulin-binding region domain containing protein.	
12	K03216	K18912	AK101795	Os04t0382300-01	Similar to SNF1-related protein kinase regulatory gamma subunit 1.	S
No independent lines available for confirmatory screening (5 lines)						
20	K17110		AK101316	Os07t0435100-01	Similar to 26S proteasome subunit RPN12.	
21	K19720		AK072674	Os03t0333300-02	Similar to eukaryotic translation initiation factor 2 beta subunit.	
24	K04020		AK066139	Os09t0461700-01	Alpha/beta hydrolase fold-3 domain containing protein.	S
26	K39531		AK099196	Os02t0590400-02	Lecithin:cholesterol acyltransferase family protein.	
31	R05018		AK111775	Os01t0313300-01	Similar to EREBP-3 protein (Fragment).	S

[1]Line No. zero is identified in this work. Other lines are derived from our previous work (Dubouzet *et al.*, 2011).

[2]RT represents retransformants and were used as independent lines.

[3]ID and predicted protein annotation provided by RAP-DB (http://rapdb.dna.affrc.go.jp/)

[4]*Rhizoctonia solani* on the FOX hunting lines in this work; R, resistant; S, susceptible

Supplementary Table S2. Primers used for quantitative real-time PCR.

Organism	Target	Forward	Reverse
Arabidopsis	*Actin2* (internal control)	CACTTGTGTGTGACAAACTCTCTGG	GGGACTAAAACGCAAAACGAAA
Rice	*Rubq1* (internal control)	GGAGCTGCTGCTGTTCTAGG	TTCAGACACCATCAAACCAGA
	BSR2	GGACTAAGACGAGGAGAGGGAAG	AACGTAGGGGCATTTCTACTCAA
	BSR2 (for checking KD level)	CCGTCGACACAGGACTG	GGCGACGAAGCCGACGAC
R.solani	*ITS3 rDNA*	GCTTCACACCTGCTCCTCTTT	CGGTTCATCTGCATTTACCTTG
C. higginsianum	*Actin*	CCGCAGACCGCAATCTT	AATGGAGGCTGAGAGCTGGTT

Supplementary Methods

Sequence alignment and phylogenetic analysis.

Amino acid sequence alignments were generated using the CLUSTAL W analysis tool from the DNA Data Bank of Japan (DDBJ; http://www.ddbj.nig.ac.jp/index-j.html) and illustrated using Bioedit (http://www.mbio.ncsu.edu/BioEdit/bioedit.html). The phylogenetic tree was constructed from the deduced amino acid sequences using the neighbour-joining method. The bootstrap mode (1000 replications) was used to estimate the confidence that could be assigned to nodes on the tree. An NJ plot[1] was used to present the data obtained.

Sheath inoculation assay for *R. solani* in rice.

The sheath inoculation assay with *R. solani* (MAFF243956) was performed as follows. Three-day-old *R. solani* grown on PDA solid medium (50 mL) were crushed using a mortar and pestle and mixed with sterile water (25 mL). Rice plants at approximately the heading stage were inoculated with this mycelial agar liquid (100 μL). The liquid was inoculated inside the auricle part of the leaf sheath of the third leaf from the flag leaf. Disease severity was evaluated 2 weeks after inoculation by measuring the length of the disease lesion in the leaf sheath of the flag leaf.

Phytohormone measurement.

Measurement of SA and JA from *Arabidopsis* plants was performed as follows. T2 seeds of the transgenic lines were sown on half-strength MS medium containing hygromycin (10 μg/mL) in a Petri dish and grown in a chamber at 22 °C under short-day conditions with 9 h of light. Approximately 3-week-old *Arabidopsis* plants exhibiting hygromycin resistance

were transferred to 60-well plates containing pre-sterilized moist peat moss (Super Mix; Sakata, Yokohama, Japan) and grown under aseptic conditions for 2 weeks. Plants were dipped for 30 s in a suspension containing *Pst* DC3000 at 10^7 cfu/mL supplemented with 0.05 % Silwet L-77 and incubated for 48 h in the dark. Aerial part of the plants was sampled, freeze-dried and their SA and JA contents were measured as previously described[2].

Ethylene released from *BSR2*-OX and vector control *Arabidopsis* plants was measured as follows. T2 seeds of the transgenic lines were sown on half-strength MS medium in a Petri dish and grown in a chamber at 22 ° C under short-day conditions with 9 h of light. Approximately 3-week-old *Arabidopsis* plants were transferred to aseptic hydroponic culture conditions in a Petri dish for 4 d before inoculation. The plants were dipped into inoculating solution with or without *Pst* DC3000. Each plant was then placed in a separate sealed glass vial at 22 ° C in the dark. The gas in each glass vial was sampled at 48 h and 72 h and analysed for ethylene as described previously[3].

References

1. Perrière, G. & Gouy, M. WWW-query: an on-line retrieval system for biological sequence banks. *Biochimie* **78**, 364-369 (1996).

2. Yoshimoto, K. et al. Autophagy negatively regulates cell death by controlling NPR1-dependent salicylic acid signaling during senescence and the innate immune response in Arabidopsis. *Plant Cell* **21**, 2914-2927 (2009).

3. Ohtsubo, N., Mitsuhara, I., Koga, M., Seo, S. & Ohashi, Y. Ethylene promotes the necrotic lesion formation and basic PR gene expression in TMV-infected tobacco. *Plant Cell Physiology* **40**, 808-817 (1999).
